# A Survey of Environmental Perception for Unmanned Ground Agricultural Machinery in Field Environments

**DOI:** 10.3390/s26134008

**Published:** 2026-06-24

**Authors:** Qian Zhang, Wenfei Wu, Mengning Liu, Lizhang Xu, Zhenghui Zhao, Shaowei Liang

**Affiliations:** 1 School of Agricultural Engineering, Jiangsu University, Zhenjiang 212013, China; 2222416015@stmail.ujs.edu.cn (W.W.); 2222416077@stmail.ujs.edu.cn (M.L.); justxlz@ujs.edu.cn (L.X.); 2 School of Electrical & Information Engineering, Jiangsu University, Zhenjiang 212013, China; zhzhao@ujs.edu.cn; 3 Energy Internet Research Institute, Tsinghua University, Beijing 100085, China; liangshaowei@mail.tsinghua.edu.cn

**Keywords:** unmanned ground agricultural machinery, environmental perception, image-based methods, point cloud processing, multimodal data fusion, agricultural robotics, autonomous agricultural vehicles

## Abstract

**Highlights:**

**What are the main findings?**

- A comprehensive review of environmental perception methods for unmanned ground agricultural machinery in field environments.
- This paper focuses on key elements in agricultural environments, namely obstacles, crop rows, and field boundaries, and systematically categorizes the corresponding perception methods into image-based, point-cloud-based, and multi-modal data fusion approaches.

**What are the implications of the main findings?**

- The advantages and limitations of different perception methods are analyzed under complex agricultural field conditions.
- Future research directions for improving robustness and adaptability of perception systems in agricultural environments are discussed.

**Abstract:**

Unmanned ground agricultural machinery is required to operate efficiently in complex and dynamic field environments, which presupposes accurate and reliable environmental perception capabilities. This requires the machinery to perceive and respond to various typical elements in both driving and operational environments, such as obstacles, crop rows, and field boundaries. This paper focuses on typical environmental elements and analyzes the environmental perception technologies used in unmanned ground agricultural machinery during field navigation and operation. First, the working principles, advantages, limitations, and application scenarios of commonly used sensors, including vision and radar sensors, are comprehensively reviewed. In addition, the critical role of multi-sensor fusion in enhancing perception robustness and adaptability is highlighted. Subsequently, this paper centers on the specific environmental elements encountered by unmanned ground agricultural machinery. From this perspective, existing perception methods are systematically categorized and reviewed across three domains: image data, point cloud data, and multimodal data fusion. The performance differences and applicable scenarios of these methods in practical applications are also analyzed. Finally, the current challenges facing environmental perception technologies for unmanned agricultural machinery are analyzed, including multi-sensor fusion complexity, the computational–real-time trade-off, and the scarcity of specialized datasets. Future development trends and potential research directions are also discussed. This review aims to provide a reference and foundation for advancing environmental perception technologies in unmanned ground agricultural machinery.

## 1. Introduction

From departing the depot to completing field operations and returning, unmanned ground agricultural machinery typically operates through several sequential stages. The machinery first leaves the depot and travels along farm tracks to the target field. After reaching the operational area, it begins executing agricultural tasks within the field. Upon completing the assigned tasks in the current field, the machinery exits the area and moves to the next target field. After all scheduled operations are finished, it returns to the depot via the farm tracks [1]. According to the characteristics of this workflow, the overall operation can be divided into two main phases: driving and operation. The driving phase mainly includes travel on farm tracks and field-to-field transfer, while the operation phase corresponds to specific in-field agricultural activities [2].

Unmanned agricultural machinery operates in two environments—driving and operating—each characterized by distinct and specific environmental elements. The driving and operation environments contain various environmental elements, including obstacles, crop rows, and field boundaries, which constitute the primary perception targets for unmanned agricultural machinery [3,4]. Unmanned machinery must perceive environment elements and adjust path planning and posture for safe, efficient task execution. Accurate environmental perception relies on both effective acquisition of environmental information and appropriate selection of perception algorithms [5]. Vision sensors provide semantic-rich images, while Radio Detection and Ranging (RADAR) supplies spatial information for unmanned agricultural machinery. In addition to these mainstream sensors, specialized auxiliary sensors are employed in specific application scenarios [6]. Multi-sensor fusion integrates complementary data from various sensors to enhance perception accuracy and robustness in complex field environments [7].

Environmental perception methods are categorized by data modality into image-based, point cloud-based, and multimodal fusion types (Table 1) [8]. Image-based methods extract environmental information from two-dimensional image data. These methods are cost-effective and rich in semantic information but are easily affected by illumination variations and lack direct three-dimensional structural information [9,10]. Point cloud-based methods obtain environmental information from three-dimensional point cloud data. These methods provide accurate spatial structural information but often involve relatively high computational complexity [11,12]. Multimodal fusion methods integrate complementary information from different sensors to improve the accuracy and robustness of environmental perception, but they generally involve higher system complexity [13].

Overall, different perception technologies exhibit distinct strengths and limitations. The appropriate selection of perception sensors and methods should therefore be guided by specific operational tasks and environmental characteristics. Systematically analyzing elements, sensors, and methods is essential for advancing unmanned agricultural machinery perception.

**Figure 1:**
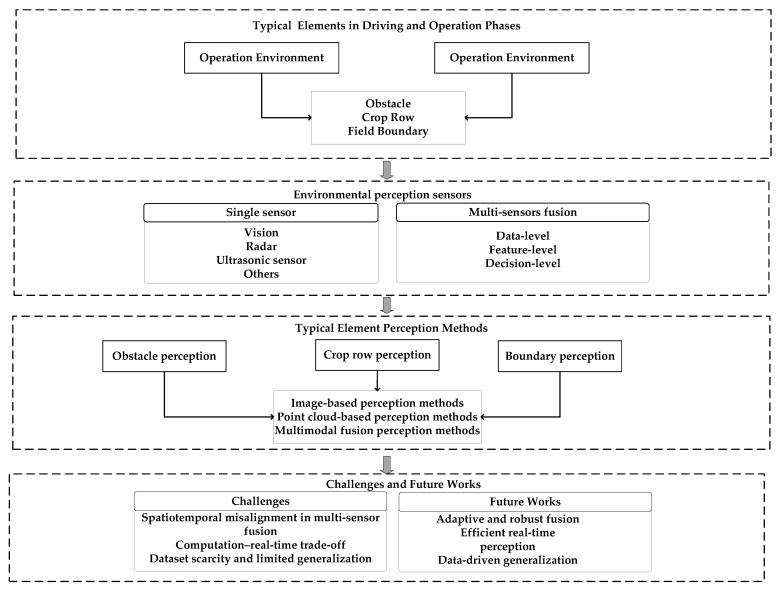
Overview of environmental perception for unmanned ground agricultural machinery.

This paper summarizes typical environmental elements, perception sensors, and multi-sensor fusion approaches for unmanned agricultural machinery. This paper focuses on the specific perception requirements for obstacles, crop rows, and boundaries during both the driving and operation of unmanned ground agricultural machinery. Accordingly, it systematically categorizes and summarizes existing perception methods across three core domains: image data, point cloud data, and multimodal data fusion. Furthermore, this paper focuses on discussing the critical issues that urgently require resolution in this field. Future development trends are outlined. The aim is to provide researchers in related fields with comprehensive technical background and development trends, offering references for future research direction. The overall framework of this review is illustrated in Figure 1.

## 2. Methodology

The literature search in this review covered six major academic databases, including the Web of Science Core Collection, Scopus, IEEE Xplore, ScienceDirect, SpringerLink, and Google Scholar. To ensure comprehensiveness and minimize omission, additional manual screening was conducted on the reference lists of retrieved papers and related review articles, so as to identify studies that may not have been captured by database queries. All retrieved records were subsequently deduplicated and consolidated.

The search period was set from January 2015 to April 2026, with the final update conducted in April 2026. This time frame was selected to capture the key developments in agricultural environmental perception and intelligent sensing over recent years, while also incorporating a limited number of seminal earlier works to preserve continuity in the technological evolution.

The study selection process followed predefined inclusion and exclusion criteria and was carried out in four stages. First, records from different databases were merged and duplicates were removed. Second, an initial screening based on titles and abstracts was performed to exclude studies that were not relevant to agricultural environmental perception tasks, such as obstacle detection, crop row detection, or field boundary detection. Third, the remaining studies were subjected to full-text assessment, and only those that explicitly addressed the target perception tasks and provided methodological details or experimental validation were included. Finally, backward reference tracking was performed to further supplement the dataset and reduce the risk of missing relevant studies, resulting in a total of 224 publications included in the final analysis.

For the analytical framework, a three-level taxonomy of “sensor–method–perception target” was established to enable structured comparison. At the sensor level, studies were grouped into visible-light sensors, infrared sensors, spectral sensors, LiDAR, millimeter-wave radar, ultrasonic sensors, and multi-sensor fusion systems, reflecting their different roles in agricultural environments. At the methodological level, approaches were categorized into image-based methods, point-cloud-based methods, and multimodal fusion methods. Image-based methods include traditional image processing techniques and deep learning approaches such as CNNs and Transformers, while point-cloud-based methods include classical geometric methods and deep learning-based models such as PointNet and PointNet++. Fusion methods were further divided into data-level, feature-level, and decision-level strategies. At the perception-target level, studies were organized into three representative tasks: obstacle perception, crop row detection, and field boundary detection, enabling consistent cross-study comparison.

In addition, a multidimensional analysis was conducted on the 213 selected publications. Keyword co-occurrence analysis and contribution-based clustering were first employed to identify major research themes, which were grouped into obstacle perception, crop row perception, and field boundary perception. Furthermore, a chronological analysis was performed to trace the methodological evolution from traditional rule-based and geometric approaches, through machine learning methods, and ultimately to deep learning-based frameworks, thereby illustrating the overall technological progression.

## 3. Typical Elements in Environmental Perception for Unmanned Ground Agricultural Machinery

The working environment of unmanned ground agricultural machinery can be broadly categorized into a driving environment and an operating environment. The driving environment mainly comprises farm tracks and the pathways connecting adjacent fields, whereas the operating environment corresponds to the interior of the target field. Each of these environments is characterized by distinct typical elements. Within the driving environment, the composition of typical elements varies with the specific travel scenario. When the machinery moves along farm tracks, the environment is primarily characterized by static obstacles, such as utility poles and stones, as well as dynamic obstacles, including field workers and other moving agricultural machinery. During field-to-field transfer, in addition to obstacles, the machinery must also contend with field boundaries formed by ridges and ditches. Under these conditions, unmanned ground agricultural machinery must perceive surrounding obstacles in real time to ensure navigation safety. Simultaneously, it is required to accurately identify field boundaries, as this is essential for enabling reliable and precise access to the designated operational areas.

Perceptual elements and task objectives for unmanned agricultural machinery vary significantly across the four operational stages: tillage, sowing, management, and harvesting. In the tillage stage, machinery performs soil plowing and leveling in bare fields containing residual debris. The operational environment at this stage is relatively simple, and the primary perception targets include field boundaries as well as obstacles such as stones, crop roots, and field residues [14]. In the sowing stage, operations are conducted on relatively flat farmland along predefined trajectories, where boundaries remain important for path planning, while obstacles still need to be detected to ensure safe navigation [15]. In the management stage, tasks like spraying, fertilization, and monitoring occur in increasingly dense crop environments. Beyond obstacles and boundaries, crop rows emerge as critical perceptual elements, serving as essential guides for precise field operations [16]. During harvesting, the presence of mature crops, lodging, and field residues heightens environmental complexity. Accurate perception of crop rows, obstacles, and boundaries becomes vital to maximizing harvesting efficiency, minimizing crop damage, and ensuring seamless coordination with transport operations [17,18,19].

**Figure 2:**
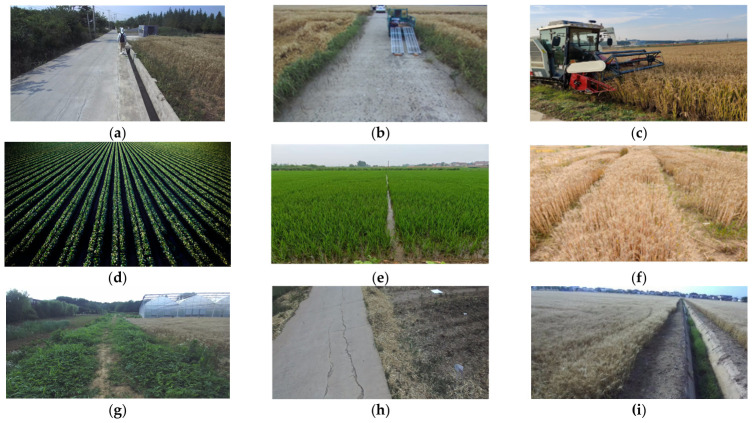
Typical environmental elements in grain transportation scenarios: (**a**) pedestrian; (**b**) vehicle; (**c**) agricultural machinery; (**d**) early-stage crop rows; (**e**) mid-stage crop rows; (**f**) mature crop rows; (**g**) field edge; (**h**) rural road edge; (**i**) ditch boundary.

Therefore, this study accounts for the typical elements found in both the driving environment and the four operational stages, namely tillage, sowing, management, and harvesting (Figure 2). Based on this comprehensive analysis, the environmental elements are categorized into three primary groups: obstacles, crop rows, and boundaries. Obstacles include both static objects, such as straw piles, stones, and utility poles, and dynamic objects, such as pedestrians and other agricultural machinery, which may appear in either the driving or operating environments. Crop rows correspond to the line-like structures formed by crops arranged with regular spacing within the operating environment. Boundaries describe the interfaces between different plots or operational areas, which are commonly associated with ridges, farm tracks, and ditches [20]. Considering that these elements are present in both the driving and operational environments of unmanned ground agricultural machinery, this paper systematically classifies and summarizes environment perception technologies for field-oriented unmanned ground agricultural machinery from the perspective of typical environmental elements, while comprehensively reviewing the associated sensing sensors and perception methods.

## 4. Environmental Perception Sensors

To ensure safe and efficient operation, unmanned ground agricultural machinery needs to perceive the key elements in its surrounding environment in real time during both driving and field operations. This capability largely depends on the appropriate selection and configuration of perception sensors. Current agricultural perception systems primarily utilize vision sensors and radars, providing image and point cloud data to represent the surrounding environment. In addition to these mainstream sensors, auxiliary sensors are often deployed to meet the requirements of specific application scenarios. In complex agricultural environments, the perception performance of a single sensor is frequently limited by factors such as restricted sensing range and susceptibility to environmental interference. To overcome these limitations, multi-sensor fusion techniques are commonly adopted. By integrating heterogeneous data obtained from different sensing modalities, multi-sensor fusion exploits the complementary characteristics of individual sensors and improves the overall accuracy and robustness of environmental perception [21]. This chapter reviews vision, radar, and auxiliary sensors in unmanned agricultural machinery, focusing on their principles, performance, and application scenarios. Furthermore, multi-sensor fusion is discussed at data, feature, and decision levels, highlighting the principles, advantages, and applications of each strategy.

### 4.1. Vision

Vision sensors obtain environmental information through the detection of optical signals and constitute one of the most important perception modalities for unmanned ground agricultural machinery. They provide essential visual cues for the perception of obstacles, crop rows, and field boundaries in agricultural environments. Commonly used sensors in unmanned agricultural machinery include monocular/stereo Red, Green, and Blue (RGB) camera, active Red, Green, Blue, and Depth (RGB-D) camera, event camera (EC), thermal imager, and hyperspectral camera. According to the spectral range of the acquired information, these sensors can be broadly classified into visible-light sensors, infrared sensors, and spectral sensors.

Visible-light sensors perceive targets primarily through color image acquisition, often in combination with image processing techniques and depth estimation methods. By capturing non-visible spectrums, infrared sensors resist illumination variations, enabling reliable nighttime and all-weather operation for agricultural machinery despite low light or glare. Spectral cameras capture multi-band spectral information, which enhances target discrimination capability in complex backgrounds. Owing to these characteristics, vision-based sensing technologies enable more stable and reliable environmental perception across diverse agricultural operational scenarios [22].

#### 4.1.1. Visible-Light Sensors

**Figure 3:**
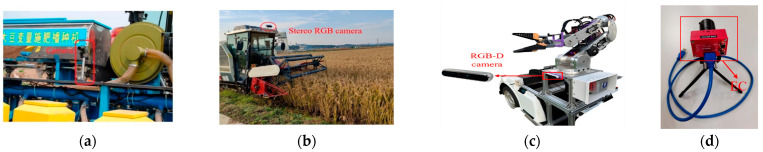
Common types of visible-light cameras used in unmanned ground agricultural machinery: (**a**) Monocular camera on a seeder (adapted from [23]); (**b**) Stereo RGB camera on a harvester; (**c**) RGB-D camera on a Fruit Picking Robot (adapted from [24]); (**d**) Event camera (EC) in a bench-top experimental setup for agricultural data collection. (adapted from [25]).

The visible light spectrum, spanning approximately 380 nm to 760 nm, corresponds to the wavelength range detectable by the human visual system. Sensors operating within this range capture scene information by recording the reflection and transmission characteristics of visible light interacting with object surfaces. As illustrated in Figure 3, typical visible-light sensors employed in agriculture include: (a) Monocular RGB camera [23]; (b) Stereo RGB camera; (c) RGB-D camera [24]; (d) EC [25].

Monocular RGB cameras are based on the pinhole camera model and the principle of perspective projection, projecting target points in three-dimensional space onto a two-dimensional image plane and establishing the mapping relationship between spatial points and image points through the camera intrinsic and extrinsic parameters [26]. In unmanned ground agricultural machinery, such cameras are commonly mounted on the front, sides, or operational platforms to provide real-time visual information for tasks including obstacle detection and crop row recognition [27]. Owing to their low cost and high spatial resolution, the monocular RGB camera is widely adopted in agricultural perception systems. However, the monocular configuration does not directly provide depth information, and depth estimation accuracy is often limited in dynamic environments. Under conditions involving high-speed motion or complex field scenes, perception performance may degrade noticeably, which poses challenges for reliable obstacle avoidance and navigation. In addition, the field of view of a single monocular RGB camera is restricted, making it difficult to cover a sufficiently wide visual area [28]. To overcome this limitation, multi-camera configurations based on multiple monocular RGB cameras have been explored to expand the effective field of view and improve perception capability. For instance, Nader et al. [29] combined multiple monocular RGB cameras with structure-from-motion (SfM) and multi-view stereo (MVS) to measure grapevine trunk diameter and volume.

Multi-view RGB camera systems estimate depth by exploiting parallax between images captured from different viewpoints, enabling three-dimensional scene reconstruction through pixel correspondence. Depending on their configurations, these parallax-based systems are typically implemented as stereo or multi-view camera architectures. In the context of unmanned agricultural machinery, stereo RGB systems featuring two viewpoints are widely adopted during field operations to provide essential depth data. By increasing the number of viewpoints, multi-view configurations can further extend the perception range and enhance depth estimation accuracy. Such systems are particularly advantageous in complex field environments or narrow passages, where richer spatial information improves maneuverability and operational efficiency under irregular terrain and obstacle-dense conditions [30]. Despite these advantages, several practical challenges remain. Stereo vision systems, the most commonly utilized configuration in this field, still face a significant computational burden, as pixel-level matching across multiple views introduces a heavy load that can compromise real-time performance, particularly at high image resolutions. Furthermore, depth estimation accuracy is inherently influenced by the baseline length; while a longer baseline improves precision, it simultaneously increases the overall system footprint. This physical constraint poses a critical trade-off, potentially hindering the maneuverability of agricultural machinery in confined spaces or during rapid motion and turns [31]. To balance these competing requirements, adjustable-baseline stereo vision systems have been investigated. For example, Yang et al. [32] utilized a high-precision sliding table and closed-loop control to dynamically adjust the stereo baseline, effectively balancing sensing accuracy with system compactness.

RGB-D cameras provide depth information in addition to conventional RGB images, enabling unmanned ground agricultural machinery to obtain explicit three-dimensional representations of the surrounding environment. In practice, RGB-D cameras are typically mounted on the front or sides of agricultural machinery to provide continuous depth data for obstacle perception and path planning [33]. According to its depth sensing principle, the RGB-D camera used in unmanned ground agricultural machinery can be broadly divided into two categories. One category is based on the structured light (SL) method. The SL-based RGB-D camera projects predefined light patterns, such as dot matrices or stripe patterns, onto object surfaces and estimates depth by analyzing pattern deformation using triangulation. With high accuracy within 0.5–5 m, these cameras excel at detecting small field obstacles like stones and weeds, enhancing collision avoidance and operational safety. However, the SL method is sensitive to strong ambient illumination and highly reflective surfaces, and depth measurement accuracy may degrade under intense sunlight or strong reflections from crop leaves [34]. The other category comprises RGB-D cameras based on the time-of-flight (ToF) method. The ToF-based RGB-D camera emits modulated infrared light and estimates target distance by measuring the time delay between signal emission and reception. Compared with the SL-based RGB-D camera, the ToF-based camera exhibits improved robustness to strong illumination and surface reflections, and it generally supports longer sensing ranges and higher frame rates [35]. ToF cameras enable large-scale perception, detecting distant obstacles in open fields and providing real-time depth data during high-speed agricultural operations. For example, Skoczeń et al. [36] developed a ToF-based obstacle detection system, demonstrating stable, long-range sensing for reliable agricultural path planning.

ECs are neuromorphic vision sensors that respond to changes in scene illumination by generating asynchronous event streams, enabling high-temporal-resolution perception of dynamic environments. Mounted on the front or top of agricultural machinery, ECs facilitate rapid detection of dynamic targets like personnel, livestock, and moving implements [37]. ECs can be used either as standalone sensors or in combination with conventional frame-based cameras, forming hybrid perception systems in which event-based data and intensity images complement each other to improve robustness. Due to their event-driven mechanism, ECs provide precise timestamped responses during rapid lighting changes or motion, enabling fast obstacle detection for agricultural machinery. Their high dynamic range and low power consumption make them particularly suitable for challenging scenarios such as direct sunlight, alternating shadow regions, and nighttime navigation. However, due to their inability to respond to static or minimally changing targets, ECs exhibit certain limitations in static agricultural environments, making it difficult to directly acquire static environmental information such as crop distribution, ground structures, and fixed obstacles [38]. Additionally, low-light or low-contrast environments reduce event generation, degrading detection accuracy and limiting reliable environmental modeling for agricultural tasks [39]. Despite these limitations, ECs exhibit distinct advantages in applications characterized by high-speed motion and strong illumination contrast. For example, Li et al. [40] combined an EC with radar sensing, where the EC captured subtle weed motion in real time while the radar provided accurate spatial localization. Field experiments demonstrated that the positioning error could be maintained within 2 cm, enabling precise weed identification and targeted treatment.

#### 4.1.2. Infrared Sensors

Infrared sensors provide essential complementary cues for agricultural perception by detecting non-visible radiation, especially under challenging lighting and environmental conditions. Agricultural infrared sensors are typically categorized into thermal imagers and near-infrared (NIR) cameras based on their spectral sensing principles.

Thermal imagers operate in the mid- to far-infrared band, typically within a wavelength range of approximately 3–14 μm, and generate images based on the thermal radiation emitted by objects. Independent of external light, these sensors enable real-time monitoring of overheating machinery components and detection of biological targets like pedestrians or animals.

In contrast, NIR optical cameras operate in the NIR range, approximately from 760 nm to 2500 nm, and acquire reflected radiation from targets illuminated by natural or artificial light sources. Leveraging spectral reflectance differences, NIR cameras enable crop growth monitoring, weed discrimination, and early detection of plant diseases or pest infestations.

Thermal and NIR sensors, typically front- or top-mounted on agricultural machinery, provide infrared data that complements visible imagery, ensuring robust path perception under varying illumination [41]. For example, Milella et al. [42] used an NIR-equipped mapping robot to capture non-visible data, supporting real-time field monitoring and path perception.

Although infrared sensors improve perception robustness in complex lighting environments, their distinct sensing mechanisms also impose specific limitations. Its detection performance largely depends on the temperature difference between the agricultural machinery operating environment and the target. When the temperature difference between the environment and the target is within ±5 K, the relative thermal radiation difference may decrease to −15% to 15%, resulting in a significant reduction in image thermal contrast [43]. In addition, thermal radiation signals can be attenuated by vegetation occlusion or moisture. NIR optical cameras, while capable of operating under low-light conditions, still depend on external NIR illumination. Their reflectance-based imaging mechanism leads to degraded performance in complete darkness, dense smoke, or scenarios where targets and backgrounds exhibit similar reflectivity characteristics [44].

Moreover, infrared imaging sensors do not inherently provide explicit three-dimensional spatial information when used alone. To address this limitation, they are often combined with complementary sensing configurations or techniques that can provide geometric cues, enabling three-dimensional perception of the operational environment and navigation path [45]. For instance, Kim et al. [46] developed a pixel-aligned RGB–NIR stereo system, spatially fusing NIR spectral data with stereo-derived geometric information to enhance agricultural perception.

#### 4.1.3. Spectral Sensors

Spectral sensors acquire information by measuring the reflectance or radiative properties of targets across multiple wavelength bands, generating spectral images that encode both spatial and spectral information. In such images, each pixel is associated with a spectral signature, which provides valuable insight into the biochemical composition and structural characteristics of observed objects. In unmanned agricultural machinery, spectral sensors are typically vehicle-mounted to capture crop and environmental data within the operational area or along navigation paths. These sensors have been widely applied in crop phenotyping tasks, where spectral reflectance data can be used to estimate key agronomic traits. For instance, Montesinos-López et al. [47] used hyperspectral reflectance to predict grain yield, effectively characterizing crop growth and biomass properties. Such capabilities highlight the potential of spectral sensing for supporting precise and large-scale agricultural monitoring.

Spectral sensors applied in agricultural machinery mainly include hyperspectral and multispectral cameras [48]. Among them, multispectral cameras usually consist of 4–10 discrete bands, primarily distributed in the visible and NIR regions. Owing to their simpler optical structure and smaller data volume, multispectral cameras can achieve faster image acquisition with relatively low computational requirements [49]. In unmanned ground agricultural machinery, multispectral sensors exploit reflectance differences between spectral bands to support basic identification of ground obstacles [50]. Additionally, NIR reflectance enables vegetation indices such as the normalized difference vegetation index (NDVI) and soil-adjusted vegetation index (SAVI) to distinguish crop-covered areas from non-crop regions, facilitating crop row identification [51].

Hyperspectral cameras consist of dozens to hundreds of narrow, contiguous spectral bands, providing much finer spectral resolution than multispectral sensors and enabling detailed discrimination of obstacle materials and crop species [52]. However, the high dimensionality of hyperspectral data substantially increases the computational burden and system cost. For real-time use, hyperspectral data requires high-performance platforms like Graphics Processing Units (GPUs) or Field-Programmable Gate Arrays (FPGAs), whose cost and complexity limit widespread agricultural deployment. As a result, hyperspectral cameras are currently more common in scientific research and high-precision agricultural management scenarios [53]. To overcome this, Gyaneshwar et al. [54] implemented a multi-class Support Vector Machine (SVM) within an FPGA-based hardware acceleration framework for direct hardware-level processing. By optimizing computational pipelines, this design enables real-time hyperspectral processing, facilitating deployment on Unmanned Aerial Vehicles (UAVs) and ground-based agricultural platforms.

Vision sensors play an important role in the perception of typical environmental elements, including obstacles, crop rows, and field boundaries. With the continuous development of sensing technologies, a variety of representative vision sensor products have emerged. Table 2 summarizes the commonly used vision sensor models and their main specifications for agricultural environmental perception.

### 4.2. Radar

Radar operates by transmitting electromagnetic waves and estimating target properties from the returned echo signals, enabling direct measurement of distance, relative velocity, and azimuth. Owing to this sensing mechanism, radar has been extensively adopted in the environmental perception systems of unmanned ground agricultural machinery. Unlike vision-based sensors, radar is independent of illumination, ensuring stable operation in low-light conditions or strong glare, such as direct sunlight [55]. Commonly deployed agricultural sensors include Light Detection and Ranging (LiDAR), millimeter-wave (mmWave) radar.

**Figure 4:**
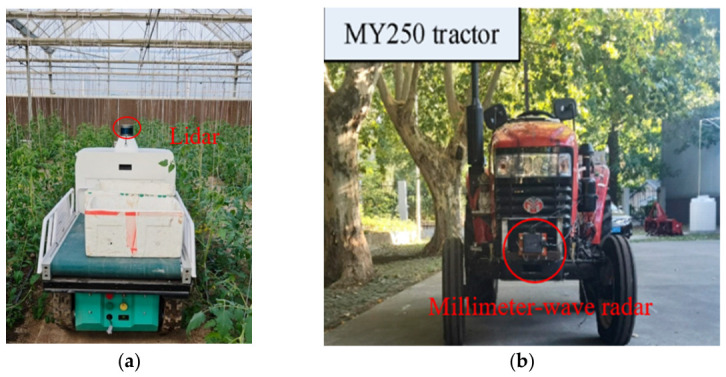
Commonly used radars on unmanned ground agricultural machinery: (**a**) LiDAR mounted on an autonomous vegetable delivery vehicle (adapted from [56]); (**b**) MmWave radar on a tractor (adapted from [57]).

LiDAR (Figure 4a) actively scans the environment with laser beams to reconstruct high-density 3D point clouds, enabling accurate obstacle detection and terrain mapping in unstructured field environments [56]. MmWave radar (Figure 4b) operates in microwave frequencies to detect long-range targets; its resilience to rain, snow, and fog ensures reliable obstacle detection and dynamic perception in agricultural scenarios [57].

#### 4.2.1. LiDAR

LiDAR transmits short NIR laser pulses (typically 905 or 1550 nm) and measures reflected signal time delays to estimate target distances. Vehicle-mounted at the front or top, LiDAR continuously scans agricultural operational areas and travel paths, providing 3D spatial information of the surroundings. Based on scanning architectures and beam steering, agricultural LiDAR is categorized into mechanical (M-LiDAR), semi-solid-state (S-LiDAR), and solid-state LiDAR (SS-LiDAR) [58].

M-LiDAR acquires environmental information by means of rotating, multi-layer laser beams, producing dense and continuous three-dimensional point cloud data. Owing to its high ranging accuracy and wide detection range, M-LiDAR enables reliable perception of field obstacles, crop rows, and terrain undulations. However, the bulky structure, high cost, and system complexity associated with rotating components limit the deployment of M-LiDAR on small platforms, restricting its current deployment to high-performance or larger agricultural machinery [59].

In contrast, S-LiDAR utilizes Micro-Electro-Mechanical Systems (MEMs) technology to control laser beam steering. Compared to M-LiDAR, S-LiDAR compact and robust structure makes it ideal for environmental perception and assisted navigation on small-to-medium agricultural machinery. However, its scanning field of view and angular resolution are generally lower than those of M-LiDAR, which limits the spatial coverage of the acquired point cloud data [60]. SS-LiDAR achieves beam scanning through purely electronic control, offering superior reliability and durability for environmental monitoring and precision navigation on small-to-medium machinery. However, its current resolution and scanning angles remain inferior to M-LiDAR, limiting perception accuracy in complex fields [61].

The technical specifications of five commonly used LiDAR models are summarized in Table 3, based on information provided in manufacturers data sheets. Despite variations in size, cost, and performance, these LiDAR configurations consistently provide accurate 3D spatial data for obstacle detection, crop row identification, and terrain perception.

The high-precision 3D data provided by LiDAR enable the perception of agricultural elements such as obstacles, crop rows, and boundaries [62]. distinguishing them based on their unique spatial distributions and geometric characteristics. For instance, isolated point clusters with distinct height variations signal obstacles, while points regularly distributed along the travel direction indicate crop rows. In addition, changes in point cloud structure at the edges of the scanned area are often used to infer the extent of field boundaries.

Based on the identification of these elements, a representation of the operational environment can be constructed, which subsequently supports path planning and autonomous task execution [63]. As an example, Xie et al. [64] proposed a LiDAR-based method for detecting negative obstacles. Their approach exploits geometric features of laser point clouds, including back-wall height, point density, and variations in point spacing, to build a mathematical detection model. By further incorporating multi-frame data fusion, the method achieved accurate detection of negative obstacles such as potholes and ditches under field conditions.

Although LiDAR provides high-accuracy three-dimensional perception, its performance is subject to several practical constraints. Under adverse weather conditions such as rain or fog, laser propagation is affected by scattering and attenuation, which degrades ranging reliability and point cloud quality. In addition, non-metallic targets such as vegetation typically exhibit low laser reflectivity, resulting in weak return signals received by LiDAR. This can degrade perception performance and make it difficult to consistently obtain reliable information about typical agricultural obstacles such as vegetation.

#### 4.2.2. MmWave Radar

MmWave radar operates in the frequency range of 30–300 GHz, corresponding to wavelengths of approximately 1–10 mm. It estimates target distance and relative velocity by transmitting electromagnetic waves and analyzing the reflected signals, with velocity information obtained through Doppler frequency shifts. In unmanned ground agricultural machinery, mmWave radar is commonly installed at the front or distributed around the vehicle body to monitor the operational area and travel path, providing real-time detection of forward obstacles. Compared with LiDAR, mmWave radar exhibits stronger penetration capabilities under rain, snow, fog, and dusty conditions, as well as higher velocity measurement accuracy. Under calibrated experimental conditions, a 77 GHz mmWave radar achieved a velocity measurement error as low as ±0.03 m/s within a speed range of 0.5–120 m/s. Furthermore, due to its relatively low cost and power consumption, mmWave radar is particularly suitable for large-scale deployment on agricultural machinery [65].

Despite its advantages, mmWave radar exhibits relatively low spatial resolution, which limits its ability to separate closely spaced obstacles, particularly when the separation distance is less than one meter. In addition, the sparse spatial sampling of radar returns restricts the construction of fine-grained environmental representations. Under complex agricultural conditions, such as irregular terrain and dense vegetation, mmWave radar measurements are also affected by multipath propagation, which can degrade ranging accuracy. Moreover, mmWave radar does not provide color or texture information, resulting in limited capability for detailed target classification. To compensate for these limitations, mmWave radar is commonly integrated with vision sensors or other perception modalities in practical agricultural applications [66]. For example, Chen et al. [67] proposed a fusion-based ranging approach that combines a monocular RGB camera with mmWave radar using an improved Extended Kalman Filter (EKF). Experimental results showed that the proposed method achieved an average close-range perception error of approximately 0.2 m, thereby improving ranging accuracy and perception robustness in complex field environments for unmanned ground agricultural machinery.

### 4.3. Ultrasonic Sensor

Ultrasonic sensor estimates target distance by transmitting high-frequency acoustic waves, typically in the range of 20–80 kHz, and measuring the time interval between signal emission and echo reception. In unmanned ground agricultural machinery, ultrasonic sensor is commonly deployed at the front, sides, or rear of the vehicle to detect nearby obstacles within the operating area, serving as a close-range sensing module for low-speed obstacle avoidance and auxiliary navigation. Owing to its short effective sensing range and centimeter-level ranging accuracy, ultrasonic sensor features a compact structure, low cost, and strong immunity to electromagnetic interference. These characteristics make ultrasonic sensor particularly suitable for applications in confined agricultural scenarios, such as narrow passages, field ends, and parking or docking maneuvers [68]. Representative ultrasonic sensor models commonly used in agricultural robots and unmanned agricultural machinery, along with their key technical specifications, are summarized in Table 4.

Despite its practicality, ultrasonic sensor exhibits several inherent limitations. The ranging accuracy of ultrasonic radar is sensitive to ambient temperature variations, with an approximate error increase of 1.7% for every 10 °C change in temperature. In addition, targets composed of sound-absorbing materials may lead to significant attenuation or loss of echo signals, which degrades obstacle detection reliability. Furthermore, slow acoustic propagation causes measurement latency, limiting ultrasonic sensor to short-range sensing and low-speed dynamic perception. As a result, in unmanned ground agricultural machinery, ultrasonic sensor is mainly employed for close-range auxiliary perception tasks, such as obstacle warning, precision operations, and low-speed navigation scenarios [69]. For example, Shreyas et al. [70] developed an obstacle avoidance system for an autonomous agricultural ground vehicle based on multiple ultrasonic sensor units. By using ultrasonic sensor for short-range obstacle detection and avoidance, the system provided reliable safety support for vehicle operation in complex farmland environments.

### 4.4. Other Sensors

In addition to the sensors discussed above, devices such as header height profiling sensors and contact sensors are relatively less adopted for environmental perception in field-based unmanned agricultural machinery. This is mainly because their sensing capability and environmental adaptability are limited, and their cost-effectiveness is comparatively lower for large-scale field perception tasks. Representative header height profiling sensors and contact sensors, along with their main specifications, are summarized in Table 5.

Header height profiling sensors measure the relative position and inclination of the header relative to the ground, enabling harvesting systems to adapt to terrain variations and maintain appropriate cutting heights. For instance, Wang et al. [71] developed a header height profiling control system for combine harvesters. Their approach fuses Global Navigation Satellite System with Real-Time Kinematic (GNSS-RTK) terrain data and Inertial Measurement Unit (IMU) attitude information. Combined with local profiling measurements, this system achieves real-time height control, enhancing harvesting stability and yield quality. Conversely, contact sensors acquire direct data from the interaction between stalks and the sensing mechanism to estimate the relative position of the harvester to crop rows. Ding et al. [72] designed an automatic row alignment platform for cotton pickers by fusing contact-sensor row deviation data with global positioning and attitude information from a GNSS/IMU system. This fusion strategy enables precise alignment of the harvesting implement with crop rows during operation.

However, the profiling accuracy of header height sensors can degrade in complex terrain, and their deployment typically involves relatively high installation and calibration requirements [73]. Conversely, contact sensors are prone to mechanical wear and crop morphological variations, potentially reducing measurement accuracy and stability while limiting their applicable scenarios during prolonged operations [74].

### 4.5. Multi-Sensor Fusion

Field environments feature volatile illumination, irregular terrain, and dynamic obstacles, challenging single-modality perception for unmanned ground agricultural machinery. Vision-based sensors, such as stereo RGB cameras, are strongly affected by illumination conditions; extreme lighting variations can degrade image quality and compromise perception reliability. LiDAR, by contrast, directly captures geometric structure through active ranging and therefore maintains stable performance under diverse lighting conditions. Nevertheless, LiDAR provides limited texture and semantic information, which constrains its ability to differentiate object categories solely based on point cloud data.

To alleviate the limitations of individual sensing modalities, multi-sensor fusion integrates heterogeneous sensor data to exploit complementary information across different perception dimensions. LiDAR provides precise spatial structures via point clouds, maintaining robustness under illumination conditions that degrade stereo RGB sensors. In contrast, stereo RGB cameras offer rich texture and semantic cues from image data, compensating for the limited semantic expressiveness of LiDAR. Fusing multi-sensor geometric and semantic data enhances perception reliability, supporting stable operation for unmanned agricultural machinery in complex field environments [75].

#### 4.5.1. Fusion Types

Current agricultural perception systems commonly fuse visible-light cameras with radar, NIR cameras, or spectral sensors to enhance unmanned machinery performance. Among these, the combination of visible-light cameras and radar has been widely investigated. Visible-light cameras provide detailed texture and semantic information but are sensitive to illumination variations. Radar sensors, in contrast, offer stable distance and spatial measurements that are largely independent of lighting conditions, although they provide limited semantic information. By integrating these complementary sensing modalities, the fused system can maintain perception performance under varying illumination while incorporating both geometric and semantic cues [76]. For instance, Lv et al. [57] presented a multi-target obstacle detection approach based on the fusion of mmWave radar and a visible-light camera. In their system, an ARS408-21 mmWave radar (Continental AG, Frankfurt, Germany) and an MCD-1073 monocular camera (Hikvision Digital Technology Co., Ltd., Hangzhou, China) were mounted at the front of an agricultural vehicle. Radar point clouds and images were transmitted to an onboard unit and jointly processed, demonstrating the feasibility of combining spatial and visual cues.

In RGB-NIR fusion, visible-light cameras provide rich texture and semantics under favorable lighting but degrade in nighttime or low-light environments. NIR cameras, which form images based on infrared radiation emitted by targets, operate independently of visible light and are therefore suitable for perception under poor visibility. However, thermal images generally exhibit lower spatial resolution and limited visual detail. This fusion integrates illumination-invariant thermal data with fine-grained visual details, enabling robust perception under varying lighting conditions [77]. Yasin et al. [78] developed an agricultural environmental perception and crop monitoring system based on the fusion of a thermal imager and an RGB camera. In their implementation, both sensors were mounted on a shared controllable platform, with data acquisition and processing performed on a Raspberry Pi-based embedded system. Registered thermal and visible images were fused in real time, generating composite representations for continuous crop and environmental perception across diverse illumination.

In spectral-radar fusion, spectral cameras provide multi-wavelength data to differentiate vegetation from non-vegetation targets like weeds or field obstacles. However, their spatial resolution is relatively limited. Radar sensors, by contrast, offer stable ranging and structural information under low-visibility conditions, while providing limited detail for target category interpretation. Integrating radar-derived spatial structures with spectral characteristics enables more effective differentiation between vegetation-related targets and non-vegetation elements [79]. Jiang et al. [80] developed a hyperspectral LiDAR system that integrates spectral sensing with laser ranging. In their system, an Acousto-Optic Tunable Filter (AOTF) is used to separate the returned laser signals by wavelength. The spectral module records reflectance intensity at different wavelengths, while the LiDAR module provides three-dimensional spatial coordinates of target points. Based on the joint use of spectral and geometric information, vegetation red-edge parameters can be extracted from the fused data, supporting crop row identification in agricultural environments.

#### 4.5.2. Fusion Methods

Multi-sensor fusion methods are generally classified into three stages based on their processing pipeline: data-level, feature-level, and decision-level fusion.

Data-level fusion integrates information directly at the raw sensor data stage. In this approach, heterogeneous sensor data are first registered via spatial alignment techniques, such as rigid transformation or the ICP algorithm. Temporal consistency is then ensured through synchronization strategies including timestamp interpolation or sliding-window mechanisms. Applying direct or weighted fusion to aligned data forms a unified dataset retaining both geometric and attribute information [81]. Because raw sensor information is directly preserved, data-level fusion is able to maintain a high level of detail, which contributes to improved perception accuracy and robustness. Bi et al. [82] presented a data-level fusion approach for constructing maize multispectral point clouds. Their method registers spectral imagery and point clouds via a Digital Surface Model (DSM), producing datasets with integrated spectral-geometric attributes. On this basis, the Rahman–Pinty–Verstraete (RPV) reflectance model is applied to describe leaf-level reflectance anisotropy, supporting crop row identification and structural analysis. However, the large data volume and the strict requirements for calibration and synchronization result in high computational cost, which limits real-time performance. As a result, data-level fusion is more suitable for applications where perception accuracy is prioritized over real-time constraints [83]. For example, the data-level fusion model RVF-Net processes up to 106 GB of data per hour. Although it achieves an AP of 54.86%, its single-frame inference latency reaches 44 ms, imposing considerable burdens on real-time computation and data transmission. Further lightweight optimization, such as network pruning and parameter quantization, is still required to reduce computational overhead [84].

Feature-level fusion integrates information by combining features extracted from preprocessed sensor data obtained from multiple sensing modalities. In this approach, feature representations from different sensors are first transformed into a common spatial reference through operations such as coordinate transformation and scale normalization. Feature association and descriptor-based matching then strengthen multimodal correspondences, creating a fused representation that incorporates complementary information [85]. Fusing feature representations rather than raw data significantly reduces data volume and computational burden, enhancing real-time performance. However, part of the original sensor information may be discarded during feature extraction, potentially affecting perception completeness. Consequently, this fusion strategy is particularly suitable for applications that require a balance between real-time performance and recognition or localization accuracy. Hu et al. [86] developed a feature-level fusion approach using a depth camera, LiDAR, and an IMU. Their system obtains maize leaf and stem texture and depth from a camera, extracts geometric features via multi-line LiDAR, and incorporates IMU data for attitude compensation. The extracted features from multiple sensors are then fused at the feature level, enabling accurate crop row identification and distance estimation in maize fields.

Decision-level fusion performs information integration at the decision output stage, where each sensor independently completes data processing and generates preliminary decision results. These decisions are combined via fusion strategies like multi-model algorithms, using dynamic or category-specific weighting to enhance final reliability and robustness. Given its simple implementation and low computational cost, decision-level fusion suits applications requiring high real-time performance and rapid dynamic responses [87]. Daneshtalab et al. [88] presented a decision-level fusion approach for agricultural land cover classification based on image and point cloud data. In their work, the classification performance of individual classifiers for different land cover categories is evaluated using metrics such as confusion matrices. Assigning category-dependent weights based on this evaluation enables a weighted voting strategy, improving overall classification accuracy.

## 5. Obstacle Perception Methods

**Figure 5:**
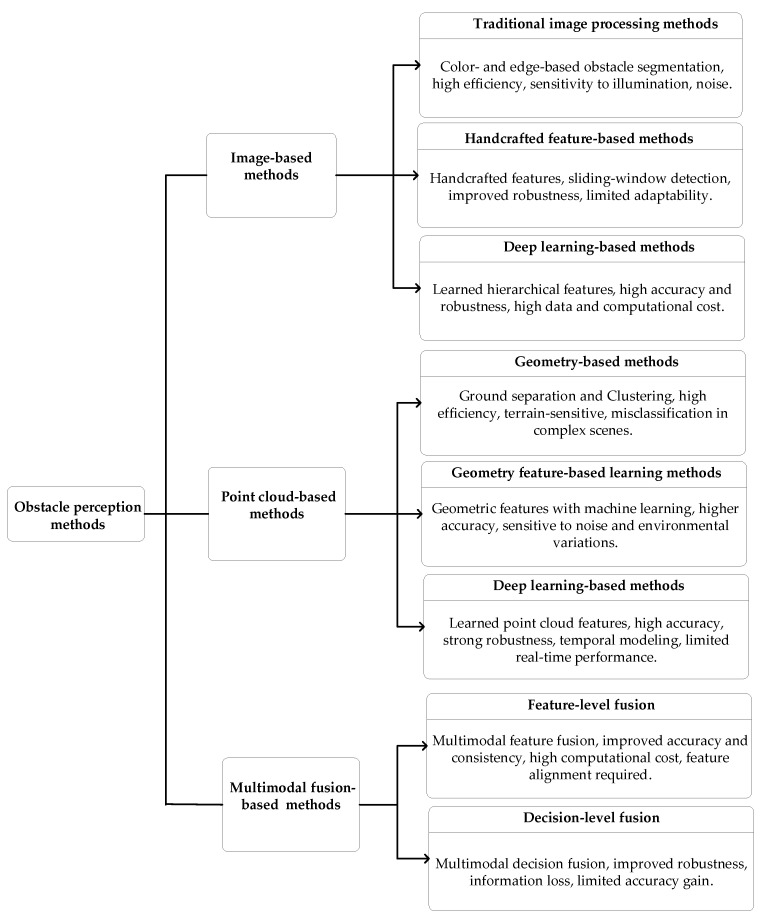
Overview of obstacle perception methods for unmanned ground agricultural machinery.

Unmanned agricultural machinery must reliably detect diverse static and dynamic obstacles, such as farm implements and pedestrians, during driving and field operations. Limited by visibility and fatigue, manual observation cannot ensure continuous, high-precision operation, making robust autonomous perception essential [89]. Existing methods can be broadly classified into image-based, point cloud-based, and multimodal data fusion approaches. Image-based methods use visual features for semantic understanding but lack explicit 3D information and are sensitive to complex environments. Point cloud-based methods provide accurate geometric representations but suffer from high computational cost and sensitivity to noise. Multimodal fusion methods combine complementary sensory information, achieving improved accuracy and robustness under varying conditions. This chapter systematically reviews these approaches, analyzing their development, advantages, and limitations to support the design of effective obstacle perception systems. To facilitate a more intuitive understanding of the classification, development, and characteristics of these methods, a schematic overview of this chapter is illustrated in Figure 5.

### 5.1. Image-Based Obstacle Perception Methods

Image-based methods leverage computer vision and machine learning to extract discriminative features like color, texture, edges, and shape cues. By analyzing pixels or regions within an image, these methods perform obstacle detection and category recognition. This paradigm has shifted from hand-crafted feature engineering to deep learning–driven frameworks, significantly enhancing perception robustness in complex agricultural settings [90].

Early image-based obstacle perception methods in agricultural environments mainly depended on traditional image processing techniques, including edge detection, morphological operations, and optical flow analysis. By extracting low-level features (color, texture, and edges) and exploiting spatio-temporal continuity, these methods effectively achieved obstacle segmentation and recognition. Motion-related cues, including image gradients and pixel intensity variations across consecutive frames, were further utilized to support dynamic obstacle analysis. As shown in Table 6, traditional image processing methods rely on low-level visual features and spatio-temporal consistency for obstacle perception. These methods are computationally efficient and suitable for real-time applications, but are sensitive to illumination changes, noise, and complex backgrounds. Their performance degrades notably when the target is visually similar to the background [91]. To enhance robustness under varying environmental conditions, Ollis et al. [92] proposed a dynamic modeling approach that combines non-parametric density estimation with image threshold segmentation. Bypassing explicit background modeling improves adaptability to lighting and occlusions, enabling more reliable dynamic obstacle detection in unstructured agricultural scenes. Nevertheless, its effectiveness remains limited when dealing with static obstacles that closely resemble the background or in the presence of severe noise interference.

Dalal et al. [99] proposed the Histogram of Oriented Gradients (HOG) descriptor, characterizing local regions via the statistical distribution of gradient orientations. By capturing edge and contour structures, HOG features enhance robustness to illumination and noise, ensuring stable recognition accuracy. Based on HOG feature representations, machine learning classifiers, most notably SVMs, were subsequently adopted for obstacle detection tasks. In such frameworks, A sliding-window strategy extracts HOG features for classifier-based determination, enabling frame-wise obstacle detection through exhaustive local region analysis. For example, Lin et al. [100] proposed a real-time obstacle detection and multi-class recognition method for agricultural environments by integrating a stereo vision system with HOG features and an SVM classifier. In their system, stereo vision was used for both image acquisition and depth estimation, achieving a distance estimation error within 1.8% for obstacles located at distances ranging from 2.5 to 20 m. However, these handcrafted features and complex training procedures hinder adaptability to dynamic obstacle categories and limit overall generalization capability [101].

Krizhevsky et al. [102] used AlexNet to outperform ImageNet benchmarks, demonstrating CNNs’ potent feature learning and accelerating deep learning adoption. Girshick et al. [103] introduced Regions with CNN features (R-CNN), integrating region proposals with CNN features to significantly outperform traditional object detection methods. Long et al. [104] proposed Fully Convolutional Networks (FCN), replacing fully connected layers to enable end-to-end semantic segmentation with superior spatial detail.

To cope with the diversity of obstacle categories and pronounced scale variations in farmland environments, Xue et al. [105] proposed an improved YOLOv5s-based detection method. Specifically, anchor box scales were redefined through K-means clustering, and the Generalized Intersection over Union (GIoU) loss was replaced with the CIoU loss in the bounding box regression stage to improve localization accuracy. The improved model increased mean Average Precision (mAP) by 5.80%, cut inference time by 75%, and outperformed Faster R-CNN in small-object detection. Zhou et al. [106] proposed an improved YOLOv8-based obstacle detection model for agricultural field environments by incorporating the CBAM attention mechanism, the BiFPN feature fusion structure, and the WIoU v3 loss function, thereby enhancing obstacle detection performance in complex farmland scenarios. The proposed method improved mAP by 1.9% compared with the original YOLOv8 while maintaining a real-time inference speed of 52 FPS. Furthermore, Wang et al. [107] developed a lightweight agricultural obstacle detection model, UAM-YOLO, by optimizing YOLOv11n with structural re-parameterization and depthwise separable convolutions. The model achieved an mAP of 95.9% while significantly reducing the number of parameters and computational cost.

**Figure 6:**
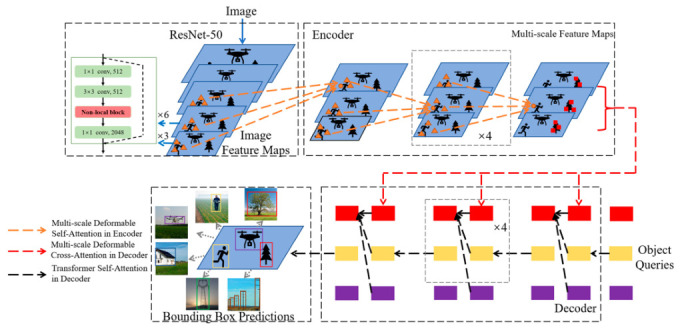
Field obstacle detection based on Non-local Deformable DETR. The colors of the boxes represent different stages of object queries in the decoder: purple indicates initial queries, yellow represents intermediate queries, and red denotes final prediction queries. The blue regions correspond to multi-scale feature maps extracted from the encoder. (reproduced from [109]).

In parallel, the Transformer architecture, originally developed for natural language processing, has been introduced into computer vision and shown strong global feature modeling capability compared with traditional CNNs. Unlike CNNs’ local receptive fields, Transformers model long-range dependencies via self-attention, better handling occlusion, scale variation, and dynamic targets in field environments [108]. Based on the Detection Transformer (DETR) framework, Wang et al. [109] proposed a field obstacle detection method using a non-local deformable DETR architecture. As shown in Figure 6, the method leverages the self-attention mechanism and non-local modules of the Transformer to enhance regional interaction, thereby improving the detection of small, slender, or occluded obstacles. The proposed method increased mAP from 71.3% to 78.0%, demonstrating superior robustness to occlusion and scale variation in field environments.

### 5.2. Point Cloud-Based Obstacle Perception Methods

Early point cloud-based obstacle perception methods were mainly based on explicit geometric modeling using height information [110]. A typical processing pipeline first separates ground points from non-ground points through plane-fitting or ground estimation techniques. After ground removal, points exhibiting a significant elevation relative to the ground plane are retained as candidate obstacle regions. These candidate points are subsequently clustered to extract obstacle contours and geometric shapes [111]. For example, Jiang et al. [112] proposed a grid-map-based obstacle detection and tracking method that integrates dynamic clustering with a Kalman filter for point cloud grouping and motion estimation. The proposed method improved accuracy by 4.86% and cut computation time by 47.8% over DBSCAN, while maintaining a tracking error under 5% for moving obstacles. Such methods are effective in scenarios where obstacles exhibit clear height differences relative to the ground, but they are prone to missed detections and false alarms when targets lack prominent height features [64].

To overcome the inherent limitations of height-based methods, subsequent studies incorporated additional geometric features, such as normal vectors, curvature, and point density. Based on these features, handcrafted descriptors are constructed and subsequently fed into machine learning classifiers, such as SVMs or Random Forests (RFs), for obstacle perception. Unlike height-difference-based rules, these feature-based approaches better capture geometric structures, improving recognition of low-height and morphologically diverse obstacles [113]. As a representative example, Ci et al. [114] proposed an obstacle detection method based on local spatial features extracted from point clouds. In this method, local point clouds are first extracted through superpixel segmentation and stereo matching. Obstacle probabilities are then estimated using normal vectors and height features, and the resulting decisions are fused within a Bayesian framework. In addition, a multi-level Stixel representation is introduced to further improve scene representation and modeling capability. On the KITTI dataset, the proposed method achieved a 96.8% True Positive Rate (TPR) with only 3.5% False Positive Rate (FPR).

However, reliance on handcrafted features limits representational capacity and generalization, hindering effective perception in complex field environments. To overcome these limitations, subsequent research has focused on more efficient feature learning strategies. In this context, deep learning-based methods were introduced into point cloud perception, benefiting from end-to-end automatic feature learning and exhibiting superior representation capability and adaptability [115]. Among these approaches, PointNet represents a seminal work in point cloud-based perception. PointNet pioneers the direct processing of point clouds as unordered sets by feeding raw point coordinates into a neural network. Symmetric functions ensure permutation invariance, while fully connected layers extract features to overcome the limitations of handcrafted designs [116]. Building upon PointNet, PointNet++ further introduces a hierarchical multi-scale grouping and feature aggregation mechanism, enabling more effective perception of structures across different scales and complex geometries. This design enhances fine-grained extraction for non-convex obstacles, improving robustness and generalization while reducing reliance on manual features. Through progressive abstraction, its hierarchical feature learning architecture enables efficient and structured understanding of point cloud data [117]. Xu et al. [118] used PointNet for feature extraction followed by RF ensemble classification, achieving 94.4% overall and 84.9% average accuracy. However, PointNet and PointNet++ still exhibit certain limitations when processing sparse point clouds. When the point cloud density is low, the geometric information available for describing object shapes and spatial structures is significantly reduced, thereby weakening feature representation capability. This issue is particularly pronounced in agricultural environments, where vegetation occlusion, variations in target distance, and non-uniform sensor sampling often result in sparse or unevenly distributed point clouds. Consequently, the representation of fine-grained structures and complex spatial relationships may be degraded, leading to reduced perception accuracy [119]. To address this issue, Zeynep et al. [120] proposed DAPNet++, which incorporates a density-adaptive strategy into PointNet++. Specifically, it dynamically partitions point cloud blocks based on local point density and adaptively determines the neighborhood search radius, thereby enhancing local feature learning in sparse and non-uniform point clouds and improving semantic segmentation performance.

**Figure 7:**
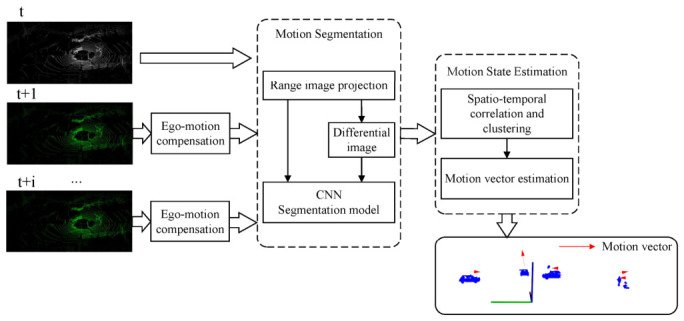
Dynamic object perception and motion state estimation using multi-frame sequential point clouds (reproduced from [122]).

In recent studies, increasing attention has been paid to temporal modeling of multi-frame point clouds for dynamic perception and continuous obstacle tracking. Such methods go beyond frame-wise obstacle identification by explicitly modeling the temporal evolution of obstacle positions. As a result, continuous perception can be maintained even under occlusion or temporary target disappearance [121]. As illustrated in Figure 7, Wu et al. [122] proposed a framework that enables continuous dynamic object perception and motion estimation in complex field environments through the integration of temporal information and spatial constraints.

### 5.3. Multimodal Data Fusion-Based Obstacle Perception Methods

Obstacle perception places stringent requirements on both response speed and detection accuracy, especially in complex and dynamic field environments. In such scenarios, perception systems must maintain fast response and stable recognition performance. Accordingly, the selection of an appropriate fusion strategy needs to jointly consider real-time performance and perception accuracy. Among the three fusion levels, data-level fusion preserves the raw information of each modality to the greatest extent but suffers from high computational cost and poor real-time performance. In contrast, feature-level fusion and decision-level fusion achieve a better balance between efficiency and accuracy. Therefore, recent studies tend to adopt feature-level and decision-level fusion to enable efficient and robust obstacle perception [123].

#### 5.3.1. Feature-Level Fusion

Feature-level fusion integrates information from different sensing modalities at the feature extraction stage, enabling the complementary characteristics of heterogeneous data sources to be jointly exploited. This strategy enhances the accuracy and robustness of obstacle perception [124]. Existing feature-level fusion methods mainly include multimodal feature encoding and fusion methods based on unified spatial representations, as well as multi-scale feature fusion techniques.

**Figure 8:**
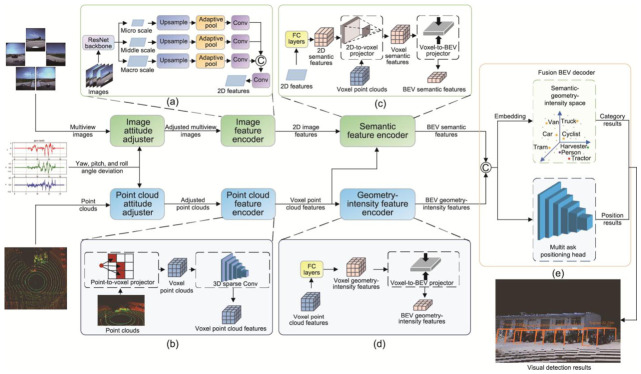
Obstacle detection architecture based on multimodal feature representation. (**a**) Image feature encoder; (**b**) point cloud feature encoder; (**c**) semantic feature encoder; (**d**) geometry–intensity feature encoder; (**e**) fusion BEV decoder. (reproduced from [126]).

Multimodal feature encoding and fusion methods based on unified spatial representations: These methods first align heterogeneous sensor data within a unified spatial representation space and subsequently perform multimodal feature encoding and fusion, thereby enabling complementary semantic and geometric information from different sensing modalities to be jointly exploited. By integrating image semantic features and point cloud geometric–intensity features within a unified representation framework, these methods improve the robustness and accuracy of obstacle perception in complex agricultural environments [125]. For example, Wang et al. [126] proposed a multimodal feature representation framework for 3D agricultural obstacle detection based on camera and LiDAR data. As illustrated in Figure 8, image and point cloud attitude adjusters are introduced to improve the consistency and reliability of multimodal inputs. Semantic and geometry–intensity feature encoders are then employed to extract heterogeneous features, while a Bird-Eye-View fusion decoder is designed to achieve unified spatial fusion and obstacle representation. Experimental results demonstrated that the proposed method achieved high detection accuracy and strong generalization capability under few-shot and zero-shot agricultural scenarios.

Multi-scale Feature Fusion: Obstacle characteristics, such as size and shape, exhibit different representations at different scales. Fusing multimodal features across multiple scales enables a more comprehensive description of obstacle diversity and structural complexity. Features extracted from shallow network layers preserve high spatial resolution and fine-grained details, which are particularly effective for detecting small-scale obstacles. In contrast, deeper network layers provide lower-resolution but semantically richer representations, contributing to more reliable recognition of large or structurally complex obstacles. By jointly leveraging information from multiple scales, this fusion strategy improves the adaptability of perception systems in complex and dynamically changing environments [127]. For example, Gao et al. [128] proposed an obstacle detection method based on Multi-Scale Selective Kernel Fusion (MSSKF), which integrates image, point cloud, and IMU data. Experimental results showed that the proposed method achieved a detection precision of 90.1% for pedestrians and agricultural machinery.

By fusing feature representations from multimodal data, including point clouds and images, these methods jointly exploit complementary and correlated information, resulting in more robust obstacle perception in complex environments.

#### 5.3.2. Decision-Level Fusion

Decision-level fusion enables accurate and timely perception and recognition of various obstacles in field environments by integrating the recognition results produced by individual sensing modalities. Common decision-level fusion methods mainly include Dempster–Shafer (D-S) evidence theory and rule-based fusion approaches [129].

D-S evidence theory fusion assigns basic probability assignments (belief values) to detection results obtained from different sensing modalities, such as images and point clouds. The Dempster–Shafer combination rule is subsequently applied to integrate uncertain or conflicting evidence across modalities, resulting in more reliable perception outcomes [130]. For example, Javadi et al. [131] applied D–S evidence theory to fuse multi-sensor data for mobile robot perception in agricultural environments. Experimental results demonstrated that the proposed method achieved improved robustness under uncertain and noisy conditions compared with conventional fusion approaches.

Rule-based Fusion Methods: Rule-based fusion methods rely on predefined decision rules to integrate recognition results from multiple sensing modalities. Such methods are commonly adopted in scenarios where obstacle characteristics and sensor reliability can be explicitly described. Fusion rules are typically formulated according to obstacle attributes, such as size, spatial location, or motion state, to determine the relative priority of different modalities. For example, when dynamic obstacles are present, perception results from modalities with higher temporal responsiveness may be assigned greater weight, while more stable modalities can be emphasized for static obstacle detection [132]. As a representative example, Prati [133] developed an interpretable fuzzy fusion framework in which SVMs are used to derive decision rules, and fuzzy logic is employed to model obstacle attributes and uncertainty. Experimental results showed that the proposed system achieved an obstacle detection accuracy exceeding 95%, demonstrating the effectiveness of rule-based fusion in structured agricultural environments.

Compared with feature-level fusion, decision-level fusion operates at a higher level of abstraction by integrating the final recognition results produced by individual sensing modalities. By combining independently derived perception outcomes, this strategy uses the complementary strengths and reliability of different sensors. Adaptive weighting schemes, such as those based on evidence theory, or rule-based reasoning informed by domain knowledge, are commonly employed to reconcile potentially conflicting decisions. As a result, decision-level fusion can provide more stable and reliable obstacle perception outcomes in complex field environments.

## 6. Crop Row Perception Methods

**Figure 9:**
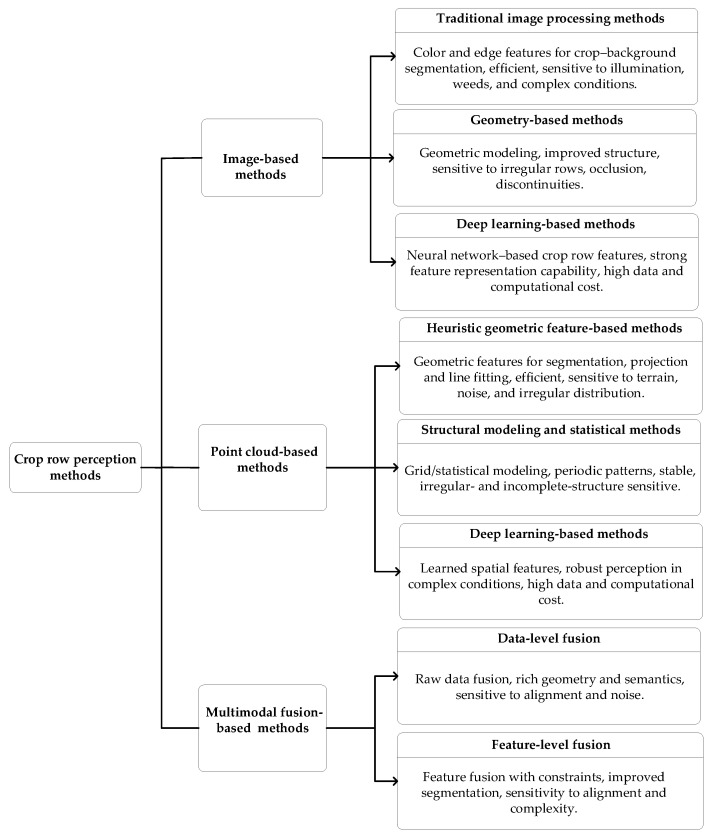
Overview of crop row perception methods for unmanned ground agricultural machinery.

Crop rows are highly regular structural elements in field environments and serve as critical navigation cues for unmanned agricultural ground vehicles. Accurate crop row perception enables reliable path planning while reducing crop damage, thereby improving both operational efficiency and crop protection performance. Existing methods can be broadly classified into image-based, point cloud-based, and multimodal data fusion approaches. Image-based methods are widely used in early growth stages due to clear crop–soil contrast and regular row structures, but their performance degrades in later stages with dense canopies, row deformation, and weed interference. Leveraging 3D geometry, point cloud methods achieve centimeter-level accuracy via clustering and fitting, addressing complex growth conditions [134]. Multimodal fusion integrates complementary sensor data, overcoming single-modality limitations and enhancing robustness in diverse field environment. This chapter systematically reviews these methods, analyzing their evolution, advantages, and limitations to support the design and optimization of crop row perception systems. To facilitate a more intuitive understanding of the classification, development, and characteristics of these methods, a schematic overview of this chapter is illustrated in Figure 9.

### 6.1. Image-Based Crop Row Perception Methods

Early image-based crop row perception methods mainly relied on fixed thresholding in color spaces such as RGB and hue–saturation–value (HSV) to segment vegetation regions, thereby roughly identifying crop row structures. While straightforward and efficient, these approaches lack robustness due to sensitivity to weeds, soil color, and illumination. For example, Romeo et al. [135] applied color threshold segmentation to separate crop vegetation from non-crop regions such as soil and stones. Despite success under uniform lighting, the method suffers false detections when non-crop areas mimic crop color.

To alleviate the limitations of methods based solely on color information, edge detection techniques such as Sobel, Prewitt, and Canny were gradually adopted in crop row perception. These approaches utilize grayscale gradient and structural cues to extract edge contours between crops and the background, enabling a clearer representation of crop row geometry and spatial arrangement. When combined with noise suppression and edge thinning operations, edge continuity and structural completeness can be improved. As a result, these methods enhance crop row detection performance in scenarios with low color contrast and reduce misclassification caused by an overreliance on single color features in complex backgrounds [136].

To improve the robustness of crop row perception under conditions such as row discontinuity and noise interference, edge detection has been combined with other image processing techniques. For instance, reference [137] presented an image-based crop row recognition method targeting discontinuous crop rows during the cotton seedling stage. The method integrates Otsu-based image binarization with Canny edge extraction, followed by morphological dilation and connected component analysis to identify crop row regions. On this basis, least squares fitting is applied to estimate crop row navigation lines, resulting in more stable and reliable crop row detection.

Driven by advancing sensing techniques, crop row detection evolved from relying on color features to incorporating color, shape, and texture cues for improved robustness [138]. Typically, crop regions are coarsely extracted via color information, then refined using shape features to validate row structures for enhanced robustness [139]. Along this line, Chen et al. [140] proposed a crop row detection method based on multi-feature fusion. Following color segmentation, the method extracts texture, shape, and orientation features for SVM-based classification, enabling precise crop row detection in complex environments.

Given the predominantly linear geometric characteristics of crop rows, Hough Transform-based line detection methods have been widely adopted for crop row perception. The Hough Transform maps line features from the image space into a parameter space, where peak responses correspond to candidate linear models of crop rows, enabling their extraction [141]. Although effective for straight crop rows, its performance degrades in scenarios involving curved rows or partial occlusion. To alleviate this limitation, Winterhalter et al. [142] proposed an improved crop row detection approach based on a patterned Hough Transform. The method decomposes crop rows with low curvature into multiple short linear segments for separate detection, thereby improving recognition performance for mildly curved rows. However, the detection accuracy remains limited when dealing with crop rows exhibiting large curvature variations. Subsequently, taking advantage of the characteristics of curved crop rows, which can be approximated as locally linear while exhibiting globally continuous curvature, researchers proposed improved methods such as the segmented Hough transform and polynomial fitting to address the aforementioned limitations. For example, García-Santillán et al. combined image partitioning, quadratic polynomial fitting, and local Hough transform to model and detect crop rows, thereby improving the detection performance for curved crop rows. The proposed method exhibited good adaptability to straight, curved, and irregularly spaced crop rows, achieving detection accuracies ranging from 86.3% to 92.8% [143].

To improve the automation level and generalization ability of crop row perception, deep learning methods, especially CNNs, have been gradually introduced into this field. CNNs adopt hierarchical network architectures composed of convolutional, pooling, and fully connected layers, and are typically trained in an end-to-end manner using large-scale annotated image datasets. Through automatic feature learning, CNNs capture crop row spatial patterns and boundaries, achieving higher accuracy and adaptability than traditional image processing [144]. For example, Osco et al. [145] proposed a CNN-based deep learning approach for the simultaneous detection and localization of planting rows. Experimental results in cornfield scenarios demonstrate that the proposed method maintains stable detection performance under different crop types and planting patterns.

On this basis, deep learning-based semantic segmentation methods have been introduced to further enhance the accuracy of crop row perception. Semantic segmentation assigns category labels to individual pixels, enabling fine-grained and accurate extraction of crop row regions [146]. For example, semantic segmentation networks such as U-Net can perform pixel-level image classification, effectively distinguishing crop regions from non-crop background areas. Post-processing, such as morphological operations and connected component analysis, is typically used to extract complete, continuous crop row contours. Guo et al. [147] proposed a crop row perception method that first models the strip-like structural characteristics of the central crop row region, thereby reducing interference from lateral leaf expansion. Semantic segmentation is then applied to perform pixel-level classification, enabling accurate discrimination between crop and non-crop regions. Finally, by integrating image post-processing techniques such as morphological operations and connected component analysis, complete and continuous crop row contours are obtained.

**Figure 10:**
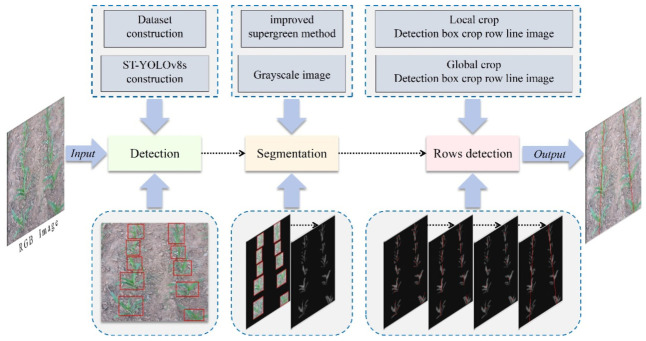
Workflow of the ST-YOLOv8s-based crop row recognition method for corn fields (reproduced from [150]).

In recent years, deep learning-based crop row detection methods have developed rapidly, and models incorporating attention mechanisms have gradually become a focus of research [148]. Among these approaches, Transformer-based network architectures have attracted considerable attention due to their advantages in global modeling. By leveraging the self-attention mechanism, these methods can capture long-range spatial dependencies, effectively overcoming the limitations of the local receptive fields of convolutional neural networks and enhancing global feature interactions. In complex agricultural environments, crop rows are often affected by occlusion, discontinuity, and background interference. Through global information modeling, Transformer architectures can better preserve structural consistency, thereby demonstrating strong robustness in related tasks [149]. As shown in Figure 10, Diao et al. [150] combined a Swin Transformer–based attention mechanism with a local–global detection strategy to improve crop row recognition in complex farmland environments. Experimental results demonstrated strong robustness and high detection accuracy across different growth stages.

### 6.2. Point Cloud-Based Crop Row Perception Methods

In the middle to late growth stages, dense crop canopies, curved row structures, and increased weed interference significantly degrade the perception performance of traditional image-based methods. By contrast, point cloud data provide explicit and continuous three-dimensional geometric information, which makes point cloud-based crop row perception an effective complement to image-based approaches [151].

Early studies on crop row perception mainly adopted heuristic height features and geometric rules, identifying crop rows by constraining plant height and spatial distribution in point cloud data [152]. A typical processing pipeline can be summarized in three main steps. First, ground points are removed using height-based segmentation to isolate crop points. Second, exploiting linear planting patterns, the method projects points onto the ground plane to estimate crop row directions via Hough Transform or least-squares fitting [153]. Based on this framework, Liu et al. [154] proposed a LiDAR-based crop row detection method. In their approach, ground points are first removed, and the remaining points are clustered to extract crop row centroids. The RANSAC algorithm is then used to fit straight lines representing crop rows. The extracted centroids and fitted line directions are further used as observations in an EKF to jointly estimate the crop row direction and the robot navigation state. These methods are simple to implement and computationally efficient. However, their performance strongly depends on parameter tuning and environmental conditions, which limits their adaptability and robustness in real-world field scenarios [155].

To enhance perception stability and structural integrity, structural modeling and statistical analysis methods have been explored. These methods typically project the point cloud onto a 2D plane and discretize it into a grid. Point density or height statistics are computed within each grid cell to construct density or elevation maps. Based on these representations, periodic patterns of crop rows can be identified using the Fourier Transform, or linear structures can be detected via template matching. In addition, geometric constraints such as row spacing and row width are often incorporated to refine crop row extraction results [156]. For example, Zhang et al. [157] generated elevation maps by projecting LiDAR point clouds onto a 2D plane. Corn rows were extracted through layered point cloud filtering, projection mapping, and rasterization. Such methods perform well under regular planting conditions but tend to yield incomplete results in cases of irregular planting or missing crop structures.

**Figure 11:**
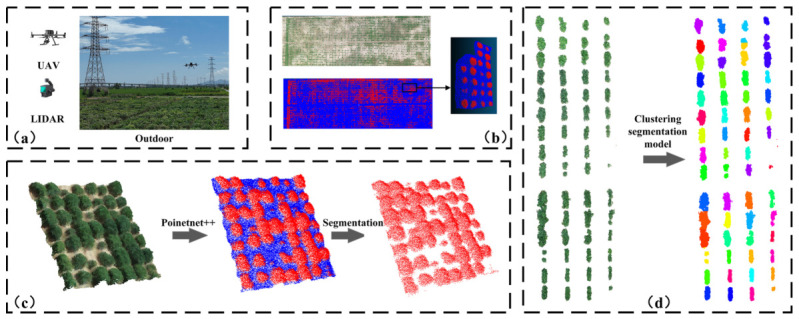
Crop row detection based on PointNet++. (**a**) data collection; (**b**) data preprocessing and dataset construction; (**c**) removal of natural background based on the PointNet++ segmentation model; (**d**) clustering-based segmentation of soybean plots. Different colors represent different instance-level clusters obtained by the clustering segmentation model. (reproduced from [160]).

To overcome these limitations, deep learning has been increasingly adopted for point cloud-based crop row perception. Through end-to-end learning on raw point clouds, deep learning models jointly capture local geometry and global spatial distributions for more expressive representations [158]. Exploiting linear and periodic crop patterns, models leverage multi-scale features to distinguish inter-row boundaries and capture complex plant morphology [159]. For instance, Shi et al. [160] employed PointNet++ to perform semantic segmentation of field point clouds, simplifying subsequent crop row extraction. As illustrated in Figure 11, the method first separates soybean plants from the surrounding environment, effectively reducing background interference. Subsequently, applying watershed and k-means algorithms segments the soybean canopy into individual plant units, facilitating subsequent crop row detection.

In recent years, increasing attention has been paid to the dynamic variations in the spatial structure of crop rows. Such structures may vary over time due to factors such as wind-induced plant motion and crop growth. As a result, single-frame point cloud data cannot sufficiently capture these time-varying characteristics, leading to reduced stability in crop row identification [161]. Accordingly, temporal information fusion techniques have been explored. These approaches integrate consecutive point cloud frames to capture spatiotemporal features associated with dynamic crop row variations [162]. Song et al. [163] addressed swaying and occlusion by employing a Transformer for point cloud registration and temporal stitching. This approach enabled continuous and dynamic perception of crop rows over time.

### 6.3. Multimodal Data Fusion-Based Crop Row Perception Methods

Crop row perception prioritizes structural accuracy and fine-grained detail over real-time performance, which has relatively lower requirements. Nevertheless, it must reliably extract highly regular crop row structures from complex and cluttered field backgrounds. Under this premise, the selection of fusion strategies should focus primarily on improving perception accuracy and structural robustness. Among the three fusion levels, data-level fusion directly integrates raw sensor data, preserving rich geometric and texture information. Feature-level fusion extracts complementary multimodal features and enhances representational capacity and accuracy. In contrast, decision-level fusion operates only on the final outputs of individual models, which may lead to the loss of fine-grained information. Therefore, existing studies more often adopt data-level and feature-level fusion [164].

#### 6.3.1. Data-Level Fusion

In crop row perception, data-level fusion typically begins with the synchronous acquisition of multiple types of raw sensor data. After modality-specific preprocessing, the raw data are fused using appropriate fusion strategies, and the resulting information is used for accurate crop row perception. This strategy aims to maximize the preservation of information from each modality, thereby alleviating the inherent limitations of relying on a single sensing source [165]. Common approaches to data-level fusion include weighted averaging and direct concatenation.

Weighted Average: This method assigns weights to raw data based on modality quality and reliability, followed by weighted average fusion to balance the contributions of different modalities [166]. For example, Sklab et al. [167] used a CNN + PointNet architecture for image and point cloud data fusion. By applying weighted fusion of 2D and 3D features, they achieved precise perception of crop row morphology.

Concatenation: This approach unifies preprocessed modalities by concatenating them along spatial, temporal, or feature dimensions to generate a joint high-dimensional dataset [168]. For instance, Wang et al. [169] first used an improved U-Net model to segment plant regions in RGB images. The segmentation results were then projected onto the point cloud, achieving spatial alignment and concatenation of semantic information with geometric data. This facilitated point cloud segmentation and cluster extraction, generating navigation lines with over 94% accuracy and under 1.45° average error.

Despite distinct characteristics, these fusion methods collectively aim to enhance crop row perception by integrating complementary multimodal data while preserving essential details.

#### 6.3.2. Feature-Level Fusion

Given their inherent regularity, crop row perception prioritizes static geometric features like spacing and alignment over the dynamic characteristics emphasized in obstacle-focused multimodal fusion. Capturing these structural cues through geometric features contributes to improved localization accuracy and more precise crop row segmentation [170]. Accordingly, feature-level multimodal fusion methods for crop row perception mainly include geometry-constrained feature fusion and region-constrained segmentation fusion.

Geometry-Constrained Feature Fusion: This approach leverages crop row geometric constraints—like parallelism and spacing—to introduce spatial priors when fusing multimodal data, such as images and point clouds. First, spatial registration and preprocessing extract candidate crop row features from images and point clouds; then, geometric rules filter and optimize these features [171]. For example, Benet et al. [172] performed multimodal fusion by combining point cloud, IMU, and image data to obtain spatial structural information of crop rows. Their method applies geometric constraints of crop rows, such as parallelism and equal spacing, to filter and optimize candidate features. This enables real-time tracking and high-precision detection of crop rows while enhancing system robustness under challenging conditions like weed occlusion or varying illumination.

Region-Constrained Segmentation Fusion: This approach introduces region-level constraints into the feature fusion of multimodal data, such as images and point clouds, to guide and refine crop row segmentation results [173]. By constraining feature fusion within consistent target regions, it improves the spatial coherence and accuracy of crop row extraction. For example, He et al. [174] proposed a multimodal crop row perception method that jointly utilizes image and point cloud data to extract the center points of rice rows. The extracted features from different modalities are spatially aligned and constrained within unified target regions, after which a robust regression algorithm is applied to fit the rice row centerlines. Based on the fitted centerlines, navigation baselines and corresponding navigation parameters are obtained. Experimental results showed that the proposed method achieved a tracking standard deviation of 27.51 mm on simulated curved rice rows.

Overall, feature-level multimodal fusion methods that explicitly incorporate crop row geometric and regional constraints effectively improve the accuracy and robustness of crop row perception in complex field environments.

## 7. Boundary Perception Methods

**Figure 12:**
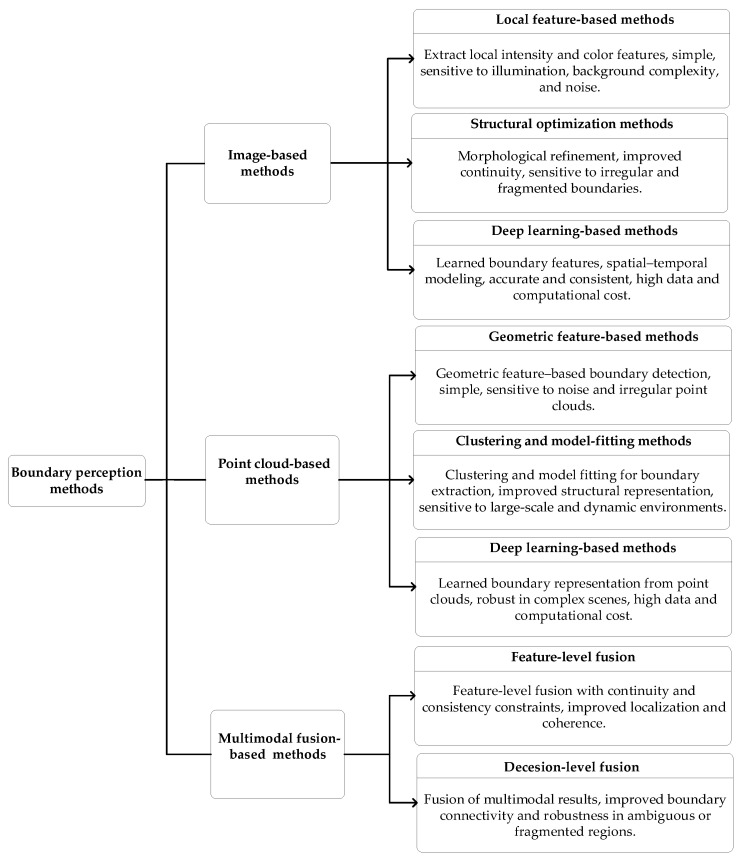
Overview of boundary perception methods for unmanned ground agricultural machinery.

In field environments, boundaries represent spatial discontinuities such as terrain features (e.g., ridges and ditches), operational structures (e.g., crop row terminations), and artificial structures (e.g., concrete embankments, field access roads). Irregular geometry, complex textures, and environmental variations make reliable boundary perception challenging, yet essential for stable path planning and precise field operations. Existing methods can be broadly classified into image-based, point cloud-based, and multimodal fusion approaches. Image-based methods extract boundary cues through edge detection, segmentation, and deep learning, offering efficient contour detection under favorable conditions but suffering in cluttered or low-light environments. Point cloud-based methods utilize three-dimensional geometric information for more accurate modeling of irregular boundaries, though they incur higher computational cost and sensitivity to noise. Multimodal fusion methods combine complementary information from images and point clouds, improving robustness and accuracy in complex environments [175]. This chapter systematically reviews these approaches, analyzing their evolution, advantages, and limitations to support the design and optimization of boundary perception systems. To facilitate a more intuitive understanding of the classification, development, and characteristics of these methods, a schematic overview of this chapter is illustrated in Figure 12.

### 7.1. Image-Based Boundary Perception Methods

Early image-based boundary perception mainly relied on traditional image processing techniques, including edge detection and region growing. Edge detection extracts boundary information by identifying abrupt intensity changes. Region growing expands regions from seed pixels based on local similarities in color or brightness, thereby achieving boundary delineation and homogeneous region segmentation [176]. Sadeh et al. [177] fused images with NDVI, used Principal Component Analysis (PCA) for dimensionality reduction, and applied Canny edge detection to extract farmland boundaries. Effective in structured environments, these local feature-based approaches remain sensitive to illumination variations, complex backgrounds, and noise interference. In addition, the implicit assumption of boundary continuity limits their performance when boundaries are discontinuous or fragmented [178].

Morphological processing has been introduced as an alternative approach to improve boundary extraction. By applying basic operations such as dilation and erosion, morphological methods suppress noise and smooth boundary structures, thereby improving boundary continuity. Unlike brightness- or color-driven techniques, these operations exploit the structural characteristics of the image, which makes boundary extraction less sensitive to illumination variations [179]. For example, Denisova et al. [180] employed NDVI-based morphological profiles for structural feature extraction and combined them with post-processing of farmland plot shapes.

**Figure 13:**
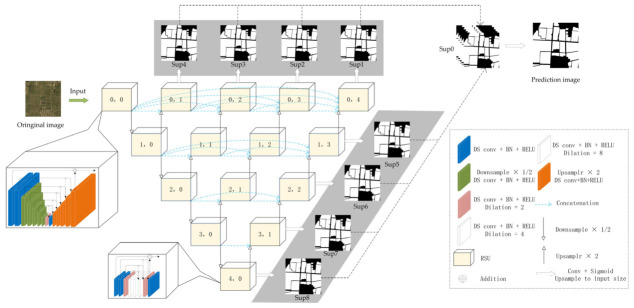
U2-Net++ network structure for farmland boundary extraction (reproduced from [183]).

Compared with conventional brightness-based segmentation methods, this approach reduced the over-segmentation rate by approximately 3% and achieved higher segmentation accuracy (Q = 0.9 versus Q = 0.85). More recently, deep learning-based approaches, particularly CNNs, have been introduced for boundary perception. By learning hierarchical features from images, these methods overcome traditional local-feature limitations, showing improved robustness in complex environments [181]. Among CNN-based architectures, U-Net has become a representative model for boundary detection. The encoder–decoder structure compresses images into high-level features, while skip connections preserve fine-grained spatial details during decoding. As a result, U-Net effectively balances global contextual information and local boundary details, reducing the occurrence of blurred or ambiguous boundary contours [182]. Building on this architecture, Chen et al. [183] proposed the U2-Net++ model for farmland boundary extraction from high-resolution remote sensing imagery. As illustrated in Figure 13, the network integrates RSU modules, depth wise separable convolutions, and channel–spatial attention mechanisms to improve the accuracy and robustness of boundary extraction.

While U-Net excels in static boundary detection, agricultural boundaries remain dynamic and vary under changing environmental conditions. Methods that rely on single images are therefore unable to capture temporal variations, which can result in unstable boundary detection. This limitation has motivated the introduction of temporal information into boundary perception. Recurrent Neural Networks (RNNs) and Long Short-Term Memory (LSTM) exploit sequential data (e.g., NDVI) by processing image sequences rather than isolated frames. These models capture temporal dependencies between adjacent frames, enabling the identification and tracking of dynamic boundary changes over time. When boundaries are partially obscured in individual frames due to illumination changes or occlusion, temporal models can further integrate information from neighboring frames to produce smoother and more consistent detection results [184]. For instance, Gao et al. [185] proposed a crop field boundary detection method based on time-varying polarization features. By incorporating spatiotemporal homogeneity measures and joint boundary strength, their approach improves both boundary detection accuracy and stability in dynamically changing field environments.

### 7.2. Point Cloud-Based Boundary Perception Methods

Early boundary detection approaches were mainly based on simple geometric rules. These methods exploit geometric information from point cloud data, including edges, corners, curves, and contours, to delineate field boundaries. In addition, after point cloud filtering, structured representations can be constructed through projection, rasterization, or voxelization, and further converted into data forms such as depth maps, elevation maps, or grid maps. This enables the application of image processing techniques, such as Canny edge detection and Hough transform, for boundary extraction [186]. Borowiec et al. [187] combined PCA with the Hough Transform on airborne LiDAR data, identifying farmland boundaries with up to 95% accuracy. Although effective in relatively simple environments, geometric feature-based methods are sensitive to noise in point cloud data. In complex field scenes, noise and irregular point distributions can degrade geometric feature extraction, resulting in reduced boundary identification accuracy [188].

More recently, clustering- and model-fitting-based methods have been explored for point cloud boundary detection and have shown advantages over traditional geometric feature-based approaches in certain scenarios. Clustering algorithms, such as DBSCAN and K-means, group point cloud data into clusters according to spatial distribution characteristics. As field boundaries often coincide with the edges of these clusters, boundary points can be inferred by analyzing cluster geometry and density distributions, particularly in scenes with relatively well-defined edges [189]. For example, Chen et al. [190] proposed a three-dimensional point cloud boundary detection method based on DBSCAN density clustering. Their approach clusters point clouds by spatial density, removes noise, and leverages local geometric features to determine boundary points. As a result, the method enables effective extraction of boundaries in complex terrain environments.

Minimum bounding box (MBB)-based methods have been introduced to better characterize the geometric form of field boundaries. Fitting minimum bounding rectangles or polygons to point clouds delineates structured boundaries and reduces false detections in dense or noisy data [191]. In parallel, surface fitting techniques have been explored to address boundary detection in more complex terrain. Fitting planes or curved surfaces to point cloud neighborhoods explicitly models boundary morphology, especially in areas with pronounced geometric variations [192]. For example, Xie et al. [193] clustered point clouds based on the coplanar condition of local quadratic surfaces (LQS) and merged surface patches that satisfied geometric continuity. In regions with geometric discontinuities, such as breaklines, their method reduced boundary segmentation error by 23.6% compared with RANSAC. Despite improvements, MBB- and surface-fitting methods struggle with accuracy and real-time performance in large-scale or dynamically changing environments.

**Figure 14:**
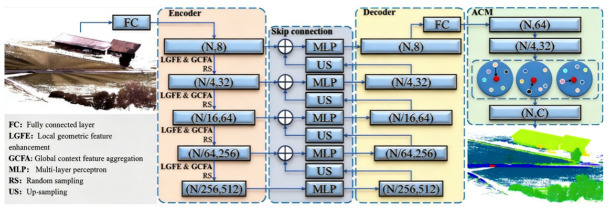
LoGA-Net for farmland boundary detection (reproduced from [195]).

Recent studies have increasingly adopted deep learning-based approaches for boundary detection from point cloud data, leading to notable performance improvements. By learning hierarchical features from large-scale point clouds, deep learning models capture local and global information, enhancing robustness across scenes. CNNs, Graph Neural Networks (GNNs), PointNet, and PointNet++ excel at processing unordered, sparse, and high-dimensional point cloud data. In boundary detection, these methods enhance accuracy and robustness against complex terrain and occlusion, supporting automated, near real-time extraction [194]. For example, Wang et al. [195] proposed LoGA-Net, a deep learning framework designed for farmland boundary detection. As illustrated in Figure 14, LoGA-Net adopts a multi-scale feature learning architecture that fuses local geometric enhancement with global context aggregation. The proposed method achieved an IoU of 78.6% in farmland boundary detection, representing a 5.9% improvement over RandLA-Net.

### 7.3. Multimodal Data Fusion-Based Boundary Perception Methods

Boundary perception aims to accurately delineate the spatial extent of operational areas by extracting reliable boundary structures. Unstructured farmland requires perception systems to maintain robustness under varying lighting, frequent occlusion, and strict real-time constraints. Under these conditions, the selection of an appropriate multimodal fusion strategy becomes critical for balancing accuracy and computational efficiency. Data-level fusion preserves raw information but incurs high computational cost. Feature-level fusion enhances the model’s ability to represent boundary information by extracting and integrating modality-specific features that are sensitive to boundary structures. Decision-level fusion improves robustness and fault tolerance in complex and irregular boundary scenarios by integrating outputs from multiple models or channels. Therefore, feature-level and decision-level fusion are more suitable for high-robustness and high-precision operational boundary delineation tasks [196].

#### 7.3.1. Feature-Level Fusion

Feature-level fusion methods operate by jointly exploiting complementary features extracted from different sensing modalities. Farmland boundary perception methods leverage contour continuity and regional consistency, imposing multi-level feature constraints to analyze boundary shape and spatial distribution. Feature-level fusion jointly optimizes multi-source features, improving localization accuracy while maintaining boundary stability and continuity under complex field conditions [197].

Feature-level fusion comprises contour-based methods emphasizing boundary continuity and region-based methods focusing on regional consistency in current boundary detection applications. Contour-based fusion exploits multi-modal edge information from images and point clouds by enforcing continuity constraints along boundary contours. Integrating multimodal edge features under contour consistency assumptions enhances complementarity and boundary robustness in complex scenes [198]. For instance, Chen et al. [199] proposed an Asymmetric Multi-modal Interaction Enhancement Network that jointly processes image and point cloud features. By introducing local edge and shape constraints within a cross-modal interaction framework, their method improves boundary perception performance in large-scale unstructured agricultural environments.

Feature fusion methods based on regional consistency focus on jointly modeling region-level features extracted from multimodal data, such as images and point clouds. These methods exploit the spatial and semantic coherence of pixels or points within the same region and introduce regional consistency constraints to guide multimodal feature integration. Constraining same-region features toward consistent representations enables effective cross-modal collaboration and stabilizes boundary feature representations [200]. For example, Cai et al. [201] proposed a Dual-Branch Spatio-Temporal Fusion Network (DSTFNet) for multimodal time-series feature fusion. Their dual-branch network integrates multimodal satellite data with regional consistency constraints, enabling accurate agricultural plot delineation.

#### 7.3.2. Decision-Level Fusion

In addition to feature-level fusion, decision-level fusion methods are widely adopted in multimodal field boundary perception tasks, particularly in scenarios with stringent real-time requirements or large-scale data processing demands. These methods perform boundary detection independently on data from different modalities and subsequently integrate the resulting decisions to obtain a unified boundary representation. A representative decision-level fusion strategy introduces spatial consistency constraints to reconcile boundary detection results across modalities. Specifically, independent boundary detection is first conducted for each modality to generate multiple candidate boundary hypotheses. Probabilistic graphical models like Conditional Random Fields (CRF) or Markov Random Fields (MRF) model spatial neighborhood consistency to capture relationships among multimodal detection results. Decision-level fusion enforces label smoothness, improving boundary closure and connectivity where modalities yield ambiguous, fragmented, or inconsistent results [202]. For example, Tuia et al. [203] proposed a multi-spatial-scale decision fusion approach based on CRF. Their method integrates multimodal classification results, jointly optimizing boundary decisions under spatial constraints to enhance coherence and robustness in complex fields.

## 8. Challenges and Prospects

Environmental perception is a core enabling technology for intelligent operation of unmanned ground agricultural machinery, as it directly supports precise, safe, and reliable task execution. With the increasing complexity and diversity of agricultural application scenarios, existing perception systems are confronted with several critical challenges.

Despite widespread use, multi-sensor fusion struggles in dynamic fields due to spatiotemporal misalignment, representation differences, and inadequate failure detection or compensation. The complexity-performance trade-off constrains deep learning deployment on resource-limited agricultural platforms, where high computational demands hinder real-time operation. Limited agricultural datasets and insufficient public coverage restrict model generalization across field environments, hindering large-scale deployment.

In summary, this chapter examines three key challenges faced by environmental perception systems for unmanned ground agricultural machinery and discusses corresponding research directions for each. Analyzing these challenges provides research insights to support the development of robust perception technologies for intelligent agriculture.

### 8.1. Multi-Sensor Fusion Challenges

Multi-sensor fusion exploits complementary information from heterogeneous sensors to overcome individual sensing limitations and enhance environmental perception. As unmanned ground agricultural machinery increasingly requires high-precision and robust perception in complex field environments, multi-sensor fusion has become a key enabling technology [204]. Despite its advantages, existing multi-sensor fusion systems still face several fundamental challenges. First, heterogeneity among sensors in terms of spatiotemporal alignment, data representation, and sampling rates limits effective information association and constrains further improvements in fusion accuracy. Many fusion frameworks lack failure-detection and compensation mechanisms, causing instability and reduced adaptability in dynamic, unstructured agricultural environments [205].

One representative solution to spatiotemporal misalignment among heterogeneous sensors is the adoption of tightly coupled filtering frameworks. Tightly coupled fusion jointly optimizes sensor states and alignment, modeling spatiotemporal correlations to enhance accuracy and robustness in dynamic environments. Liu et al. [206] tightly coupled radar and inertial data, improving accuracy by nearly 20% through reduced alignment errors. In contrast, to enhance system stability under sensor degradation or missing modalities, another line of research focuses on fusion architectures that relax coupling constraints and emphasize robustness to abnormal measurements. Simanek et al. [207] proposed an EKF-based loosely coupled fusion algorithm that filters individual sensor measurements before decision-level fusion. This design reduces computational complexity and improves fault tolerance by avoiding strong interdependencies among sensor modalities.

Tightly and loosely coupled fusion perform well in controlled settings but struggle in unstructured field environments. Tightly coupled filtering requires precise calibration, yet vibrations and disturbances cause parameter drift, hindering stable, long-term automatic maintenance. Loosely coupled fusion prioritizes robustness and low complexity over accuracy, typically underperforming tightly coupled approaches. Moreover, the relaxed coupling strategy limits the exploitation of high-frequency inter-sensor interactions, thereby constraining the ability of the system to respond rapidly to dynamic changes in complex field environments.

Based on the above analysis, future research on multi-sensor fusion for unmanned ground agricultural machinery is likely to focus on several key directions. Adaptive online calibration must dynamically compensate for sensor drift caused by vibrations and environmental disturbances in field conditions. Lightweight hybrid frameworks should integrate tight and loose coupling to balance fusion accuracy with computational efficiency and real-time performance.

In addition, data-driven intelligent anomaly detection and fault-tolerant mechanisms are expected to play an increasingly important role in enhancing system robustness against complex and unpredictable sensor failures or abnormal measurements.

### 8.2. Conflict Between Computational Power and Real-Time Performance

In complex and dynamic field environments, unmanned agricultural machinery is required to process large volumes of multi-sensor data in real time to ensure reliable perception and timely decision-making. Onboard hardware constraints hinder deploying computationally intensive algorithms, challenging real-time efficiency and operational performance in practical agricultural systems [208].

To address this challenge, researchers have adopted various strategies. For hardware, high-performance GPUs and TPUs are employed to accelerate computation, and their architectures are optimized for unmanned agricultural machinery to improve processing efficiency [209]. For example, NVIDIA’s Jetson series GPUs have been widely deployed in unmanned agricultural machinery, where their powerful parallel computing capabilities significantly accelerate sensor data processing [210]. For software, lightweight models such as MobileNet and SqueezeNet, together with model compression techniques, are utilized to reduce computational requirements and improve operational efficiency. Assunção et al. [211] simplified DeeplabV3-MobileNet, using multi-scale fusion and attention to lower computational costs without sacrificing accuracy.

Despite recent progress, several limitations remain in addressing the computational and energy constraints of unmanned ground agricultural machinery. On the hardware side, continuous operation over large-scale field environments imposes stringent requirements on reliability, stability, and energy efficiency. Embedded platforms accelerate perception but increase power and heat, challenging the balance between computational performance and energy efficiency [212]. Lightweight models reduce computational load but lack representational capacity, potentially causing perception errors that compromise operational precision and safety. Existing studies lack hardware–software co-design, focusing on single-level optimization and leaving the trade-off between computational capability and real-time performance unresolved [213].

Based on the above discussion, future research on computational efficiency and real-time perception in unmanned ground agricultural machinery is expected to focus on several complementary aspects. Dedicated accelerators and optimized heterogeneous architectures can enhance computational density and energy efficiency for long-term field operations. From a software perspective, more efficient deep learning network architectures and training strategies are needed to better balance perception accuracy with computational and energy constraints under practical deployment conditions. Hardware–software co-design is a critical direction for building efficient perception systems that ensure reliable, stable operations in complex field scenarios.

### 8.3. Scarcity of Datasets

Limited scale and diversity in public agricultural datasets further constrain environmental perception for unmanned ground machinery. The existing agricultural datasets are limited in quantity, here is a lack of public datasets that cover different crop types, growth stages, field environments and agricultural operation scenarios [214]. In addition, most current datasets have relatively small scales. Since they are mostly collected from a small number of experimental fields and specific regions with insufficient samples, these datasets fail to fully reflect the complex variations of crops, growth environments, terrain, illumination and weather conditions in real agricultural operations. For example, Agri-Point primarily provides three-dimensional geometric information on crops and ground structures, offering data support for tasks such as field environment modeling and boundary detection. However, its dataset scale is relatively limited and lacks sufficient scene diversity, making it difficult to comprehensively cover variations in crop growth conditions, ground structures, and environmental factors in complex farmland environments, thereby restricting the generalization capability of trained models [215]. The ROSE Dataset is designed for multimodal environmental perception in outdoor agricultural robotics and covers a variety of complex natural field scenarios, which helps improve the robustness and generalization ability of models in real agricultural environments. Nevertheless, its data distribution remains imbalanced in terms of crop types, terrain conditions, and acquisition regions, which may limit model transferability and generalization across different agricultural scenarios [216]. These limitations substantially hinder both the development and practical deployment of environmental perception technologies for unmanned ground agricultural machinery. Limited data scale and diversity impair model generalization, causing performance variability and reducing system stability across environments. Insufficient annotated data hinders algorithm validation, limiting further improvements in perception accuracy and robustness [217].

To mitigate the limitations imposed by insufficient data scale and diversity, existing research has mainly explored two complementary strategies. Data synthesis and augmentation, including GANs, expand datasets to improve model robustness against varying illumination and environmental changes [218]. Fawakherji et al. [219] combined GAN-based generation with conventional augmentation to expand crop and weed datasets significantly. Another research direction emphasizes the design of lightweight model architectures that are more suitable for small-sample scenarios while retaining competitive perception accuracy. Lu et al. [220] fused multi-modal data via a Transformer, reducing model size to 30% using knowledge distillation.

Existing approaches based on generative data augmentation and lightweight model design have partially mitigated the challenges posed by limited agricultural datasets and cross-scenario generalization. Limitations persist, including synthetic-to-real gaps, weak generalization, high deployment costs, and low automation in data annotation [221].

Future research can be further advanced in the following aspects. First, domain adaptation and adversarial learning can reduce the distribution gap between synthetic and real-world data. However, variations in illumination conditions, crop types, and data acquisition settings in complex agricultural environments can still lead to significant domain shifts. Therefore, improving the ability of models to adapt to domain discrepancies remains an important research direction [222]. Second, open and shared multimodal agricultural data platforms have the potential to facilitate large-scale collaborative research across different regions, crop types, and environmental conditions. However, inconsistencies in data formats, annotation standards, and cross-dataset alignment methods currently limit the sharing and utilization of data resources. Therefore, establishing unified data standards and efficient data collaboration mechanisms is a key direction for future development [223]. Third, federated learning provides a promising solution for distributed collaborative training in agricultural scenarios, enabling privacy-preserving model development without the need for centralized data collection. However, due to the wide geographical distribution of agricultural production environments, the diversity of data acquisition entities, and variations in network infrastructure, issues such as device heterogeneity, computational resource disparities, and unstable communication continue to hinder its practical deployment. Therefore, improving the training efficiency and system stability of federated learning remains a major focus of future research [224].

## 9. Conclusions

Focusing on three typical elements within the working environment of unmanned ground agricultural machinery—obstacles, crop rows, and field boundaries—this paper reviews environment perception technologies for unmanned ground agricultural machinery from two dimensions: environment perception sensors and perception algorithms for typical environmental elements, and analyzes existing challenges as well as future development trends.

First, this paper sorts out typical environmental elements encountered by unmanned ground agricultural machinery across four farming stages (tillage, sowing, crop management, and harvesting) in both driving and operation scenarios. Next, it summarizes the technical characteristics and representative applications of perception sensors including cameras, LiDAR, millimeter-wave radar, and ultrasonic sensors. The advantages and disadvantages of each sensor are compared in terms of perception accuracy, environmental adaptability, and cost, and the critical value of multi-sensor fusion for improving the accuracy and robustness of environment perception is elaborated. Targeting the three core environmental elements (obstacles, crop rows and field boundaries), this paper systematically reviews environment perception methods based on images, point clouds and multimodal fusion. It traces the evolution of image-based perception pipelines: from conventional image processing, to traditional machine learning relying on handcrafted features, and finally to end-to-end perception driven by deep learning. For point cloud-based perception approaches, this paper generalizes their technical roadmap: geometry rule-driven algorithms, machine learning methods using local geometric descriptors, and deep learning frameworks that automatically learn feature representations from raw point cloud data. In addition, fusion strategies at the data, feature and decision levels are summarized, alongside their applications in perceiving typical agricultural environmental elements.

Although remarkable progress has been achieved in existing environment perception technologies, practical deployment still faces prominent challenges, including difficult multi-sensor calibration, the trade-off between perception accuracy and real-time performance, and the scarcity of agricultural datasets. Future research should further enhance the adaptive capability of multi-sensor fusion, develop lightweight environment perception models, and promote the construction of agricultural data resources.

## Figures and Tables

**Table 1:** Comparison of environmental perception methods.

Methods	Sensors	Principles	Performances	Characteristics	References
Image-based	RGB camera, Near-Infrared camera, Spectral camera	Detection based on 2D image feature extraction.	Traditional methods (e.g., Canny) achieve an mAP of 71.3%, deep learning methods (e.g., DETR) reach 78.0%.	Rich semantics and textures, flexible deployment, illumination-sensitive, no reliable depth.	[9,10]
Point Cloud-based	LiDAR, Millimeter-wave radar	Detection based on 3D geometric information from point cloud.	Traditional methods (e.g., Euclidean clustering) achieve TPR = 96.8%, Deep learning methods (e.g., PointNet) achieve accuracy 94.4%.	Accurate 3D geometry, strong spatial awareness, lighting-robust, high cost, sensitive to sparse/noisy data, limited semantics.	[11,12]
Multi-modal Fusion	RGB camera and LiDAR	Multi-source fusion at data/feature/decision level for joint detection.	Data-level methods (e.g., concatenation) achieve accuracy > 94% with angle error ≤ 1.45°, Feature-level methods (e.g., MSSKF) achieve precision 90.1%, Decision-level methods (e.g., rule-based) achieve accuracy > 95%.	Enhanced robustness and accuracy, complex system, strict calibration and synchronization, high computational cost.	[13]

**Table 2:** Models and Characteristics of commonly used visual sensors.

Category	Model	Wavelength	Specifications	Frame Rate	Characteristics
Monocular RGB camera	Basler acA1920-40gc (Basler AG, Ahrensburg, Germany)	400–700 nm	1920 × 1200, 1280 × 720, 640 × 480	15–40 fps	High-resolution color imaging, stable imaging performance, low noise, no direct depth sensing, sensitive to illumination changes.
Stereo RGB camera	ZED 2i (Stereolabs, Paris, France)	400–700 nm	2208 × 1242, 1920 × 1080, 1280 × 720	15–100 fps	Synchronized left–right image acquisition, passive depth estimation, reduced depth accuracy with increasing target distance.
RGB-D camera	Intel RealSense D455 (Intel Corporation, Santa Clara, CA, USA)	RGB: 400–700 nm; Depth: 850 nm IR	RGB: 1280 × 800, 640 × 480; Depth: 1280 × 720, 640 × 480	≤90 fps	Simultaneous color and depth acquisition, high short-range accuracy, susceptibility to ambient light interference, limited sensing range.
Event camera	DAVIS346 (iniVation AG, Zürich, Switzerland)	400–900 nm	346 × 260	>1000 (equivalent)	Microsecond-level temporal resolution, high dynamic range, suitability for high-speed dynamic scenes, relatively low spatial resolution.
Thermal imaging camera	FLIR Boson 640 (Teledyne FLIR LLC, Wilsonville, OR, USA)	7.5–13.5 μm	640 × 512; NETD < 50 mK	60 fps	Independent of visible illumination, temperature distribution capture, low spatial resolution, dependence on temperature contrast.
Hyperspectral camera	Specim IQ (Specim, Spectral Imaging Ltd., Oulu, Finland)	400–1000 nm	512 × 512; 204 bands; spectral resolution 7 nm	Snapshot	Rich spectral information for target recognition and material analysis, large data volume, high computational complexity, high cost.

**Table 3:** Technical Specifications of Commonly Used LiDAR Systems.

Model	Parameters	Category
Velodyne VLP-16 (Velodyne Lidar Inc., San Jose, CA, USA)	16 beams/channels, Measurement range: 0.3–150 m, Field of View (FOV): 360° × 30°, Ranging accuracy: ±2 cm, Angular accuracy: 0.1°, Point cloud density: 300,000 points/s.	M-LiDAR
Ouster OS1 (Ouster Inc., San Francisco, CA, USA)	64 beams/channels, Measurement range: 0.3–120 m, Field of View (FOV): 360° × 30°, Ranging accuracy: ±2 cm, Angular accuracy: 0.1°, Point cloud density: 1,000,000 points/s.	M-LiDAR
Livox Mid-40 (Livox Technology Company Limited, Shenzhen, China)	40 beams/channels, Measurement range: 0.3–260 m, Field of View (FOV): 360° × 40°, Ranging accuracy: ±2 cm, Angular accuracy: 0.1°, Point cloud density: 480,000 points/s.	SS-LiDAR
RoboSense M1 (RoboSense Technology Co., Ltd., Shenzhen, China)	Consists of 5 laser units. Each sector has a relatively large FOV (>20°), which are combined to achieve a wide horizontal FOV.	S-LiDAR
DJI Livox Mid (Livox Technology Company Limited, Shenzhen, China)	Employs a dual-wedge prism scanning scheme. As the two prisms rotate at different speeds, they generate a flower-like scanning pattern in front.	SS-LiDAR

**Table 4:** Common Ultrasonic Sensor Models and Characteristics.

Model	Range	Frequency	Resolution	Characteristics
MB7389 HRXL-MaxSonar-WR (MaxBotix Inc., Brainerd, MN, USA)	0.3–5.0 m	42 kHz	1 mm	High measurement accuracy, strong noise immunity, supports multiple output interfaces
UC2000-30GM-IU-V1 (Pepperl+Fuchs SE, Mannheim, Germany)	0.12–2.0 m	200 kHz	0.18 mm	Industrial-grade distance measurement, high stability, strong environmental adaptability
T30UX Series (Banner Engineering Corp., Minneapolis, MN, USA)	0.2–8.0 m	44–50 kHz	1 mm	Large measurement range, excellent dustproof and waterproof performance
UM30 Series (SICK AG, Waldkirch, Germany)	0.18–8.0 m	200 kHz	0.18 mm	Fast response speed, supports real-time distance measurement

**Table 5:** Common Header Profiling and Contact Sensors: Models and Characteristics.

Model	Type	Range	Resolution	Output	Characteristics
KPM12 (ifm electronic gmbh, Essen, Germany)	Header profiling angle sensor	±90°	0.1°	Analog	Quick reaction, terrain following, angle detection, height stability
RSC-2800 (Novotechnik Messwertaufnehmer OHG, Ostfildern, Germany)	Header profiling displacement sensor	0–500 mm	1 mm	Analog	Clearance monitoring, measurement stability, automatic profiling
TSM-01 (ifm electronic gmbh, Essen, Germany)	Touch-lever contact sensor	0–300 mm	1 mm	Switch	Contact sensing, lever detection, simple structure, low cost
FSR400 (Interlink Electronics Inc., Irvine, CA, USA)	Force-sensitive contact sensor	0–100 N	0.1 N	Analog	Contact force sensing, boundary identification, inter-row positioning, high sensitivity

**Table 6:** Traditional Image Processing Methods.

Methods	Principles	Strengths and Limitations	References
Edge Detection	Identification of regions with significant intensity gradients.	Reduced redundancy, high efficiency noise and illumination sensitivity, degraded performance in cluttered or low-texture scenes.	[91,92]
Color Feature Threshold Segmentation	Regions are segmented by applying thresholds on color features.	High efficiency, real-time suitability, lighting sensitivity, degraded performance with similar foreground–background colors.	[93,94]
Morphological Processing Algorithm	Image structures are refined using morphological operations.	Geometric structure enhancement, structuring element dependence, loss of fine details in complex environments.	[95,96]
Optical Flow Method	Motion is estimated based on pixel-wise displacement using optical flow.	Explicit motion cues, dynamic scene analysis and moving object detection, high computational cost, sensitivity to noise and illumination.	[97,98]

## Data Availability

No new data were created or analyzed in this study.

## Abbreviations

The following abbreviations are used in this manuscript:

RADAR	Radio Detection and Ranging
CNN	Convolutional Neural Network
RGB	Red, Green, and Blue
RGB-D	Red, Green, Blue, and Depth
EC	Event Camera
SfM	Structure from Motion
MVS	Multi-View Stereo
SL	Structured Light
ToF	Time of Flight
NIR	Near-Infrared
NDVI	Normalized Difference Vegetation Index
SAVI	Soil-Adjusted Vegetation Index
GPU	Graphics Processing Unit
FPGA	Field-Programmable Gate Array
UAV	Unmanned Aerial Vehicle
LiDAR	Light Detection and Ranging
mmWave	Millimeter-Wave
M-LiDAR	Mechanical LiDAR
S-LiDAR	Semi-solid-state LiDAR
SS-LiDAR	Solid-State LiDAR
MEM	Micro-Electro-Mechanical System
EKF	Extended Kalman Filter
GNSS-RTK	Global Navigation Satellite System with Real-Time Kinematic
IMU	Inertial Measurement Unit
AOTF	Acousto-Optic Tunable Filter
ICP	Iterative Closest Point
RPV	Rahman–Pinty–Verstraete
HOG	Histogram of Oriented Gradients
SVM	Support Vector Machine
R-CNN	Regions with CNN
GIoU	Generalized Intersection over Union
mAP	mean Average Precision
FCN	Fully Convolutional Network
DETR	Detection Transformer
DBSCAN	Density-Based Spatial Clustering of Applications with Noise
RF	Random Forest
TPR	True Positive Rate
FPR	False Positive Rate
CRF	Conditional Random Field
MSSKF	Multi-Scale Selective Kernel Fusion
D-S	Dempster–Shafer
HSV	Hue, Saturation, and Value
PCA	Principal Component Analysis
RNN	Recurrent Neural Network
LSTM	Long Short-Term Memory
MBB	Minimum Bounding Box
LQS	Local Quadratic Surfaces
GNN	Graph Neural Network
DSTFNet	Dual-Branch Spatio-Temporal Fusion Network
MRF	Markov Random Field
